# EBF2 Links KMT2D‐Mediated H3K4me1 to Suppress Pancreatic Cancer Progression via Upregulating KLLN

**DOI:** 10.1002/advs.202302037

**Published:** 2023-11-28

**Authors:** Bing Yao, Mengying Xing, Shixin Meng, Shang Li, Jingwan Zhou, Ming Zhang, Chen Yang, Shuang Qu, Yucui Jin, Hongyan Yuan, Ke Zen, Changyan Ma

**Affiliations:** ^1^ Department of Medical Genetics Nanjing Medical University 101 Longmian Avenue Nanjing 211166 China; ^2^ The State Key Laboratory of Pharmaceutical Biotechnology School of Life Sciences Nanjing University 163 Xianlin Avenue Nanjing 210023 China; ^3^ School of Life Science and Technology China Pharmaceutical University 639 Longmian Avenue Nanjing Jiangsu 211198 China; ^4^ Department of Oncology and Lombardi Comprehensive Cancer Center Georgetown University Medical Center Washington DC 20007 USA; ^5^ Jiangsu Key Laboratory of Xenotransplantation Nanjing Medical University 101 Longmian Avenue Nanjing 211166 China

**Keywords:** EBF2, H3K4me1, KLLN, KMT2D, pancreatic cancer

## Abstract

Mono‐methylation of histone H3 on Lys 4 (H3K4me1), which is catalyzed by histone‐lysine N‐methyltransferase 2D (KMT2D), serves as an important epigenetic regulator in transcriptional control. In this study, the authors identify early B‐cell factor 2 (EBF2) as a binding protein of H3K4me1. Combining analyses of RNA‐seq and ChIP‐seq data, the authors further identify killin (KLLN) as a transcriptional target of KMT2D and EBF2 in pancreatic ductal adenocarcinoma (PDAC) cells. KMT2D‐dependent H3K4me1 and EBF2 are predominantly over‐lapped proximal to the transcription start site (TSS) of KLLN gene. Comprehensive functional assays show that KMT2D and EBF2 cooperatively inhibit PDAC cells proliferation, migration, and invasion through upregulating KLLN. Such inhibition on PDAC progression is also achieved through increasing H3K4me1 level by GSK‐LSD1, a selective inhibitor of lysine‐specific demethylase 1 (LSD1). Taken together, these findings reveal a new mechanism underlying PDAC progression and provide potential therapeutic targets for PDAC treatment.

## Graphical abstract

EBF2 is a novel H3K4me1‐binding protein. EBF2 and KMT2D cooperatively regulate H3K4me1, leading to epigenetically activation of KLLN expression and strongly inhibition of PDAC progression. Such inhibition on PDAC progression is also achieved through increasing H3K4me1 level by GSK‐LSD1, a selective inhibitor of LSD1. These findings reveal a novel pathologic mechanism underlying PDAC progression and provide potential therapeutic targets for PDAC treatment.

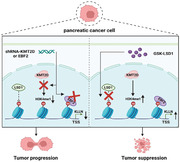

## 1 Introduction

PDAC is a highly aggressive and refractory disease with a poor 5‐year survival.^[^ ^1^ ^]^ According to the Global Cancer Statistics 2020, a total of 495773 new cases and 466003 deaths were reported, ranking PDAC the 14th in incidence and 7th in mortality (4.7% of all cancer‐caused deaths) globally.^[^ ^2^ ^]^ PDAC may arise from multiple triggers, including tobacco smoking, diabetes mellitus, obesity, dietary factors, alcohol abuse, ethnicity, family history, and genetic factors,^[^ ^3^, ^4^ ^]^ but its pathogenic mechanisms remain incompletely understood.

Covalent modifications on histone tails play fundamental roles in regulating chromatin structure and controlling transcriptional activity.^[^ ^5^, ^6^ ^]^ Histone lysine methylation has been established as a central modification in the epigenetic regulation of eukaryotic genomes.^[^ ^7^, ^8^, ^9^ ^]^ During this modification, the lysine residues on histones H3 or H4, such as H3K4, H3K9, H3K27 and H4K20, are mono‐, di‐ or tri‐methylated.^[^ ^10^ ^]^ Emerging evidence has highlighted that histone lysine methylation is dynamically regulated by both histone lysine methyltransferases (KMTs) and demethylases (KDMs).^[^ ^11^, ^12^, ^13^, ^14^, ^15^, ^16^, ^17^ ^]^ Catalyzed by KMT2D and removed by LSD1,^[^ ^18^, ^19^ ^]^ H3K4me1 serves as a powerful epigenetic component during transcriptional control. Genome‐wide ChIP‐seq analysis has uncovered that H3K4me1 predominantly deposits at a large set of distal enhancers or promoters, with a highly dynamic correlation with cell‐type‐specific gene expression profiles.^[^ ^20^, ^21^, ^22^ ^]^ H3K4me1 function as a binding site for epigenetic regulators or factors associated with gene transcription, including chromatin remodelers, histone acetyltransferases, and transcription factors.^[^ ^21^, ^23^, ^24^ ^]^ Yu et al. have found that H3K27me3‐H3K4me1 transition plays a key role in lineage differentiation by regulating the expression of tissue specific genes.^[^ ^25^ ^]^ Moreover, H3K4me1 and H3K27ac have been reported to initiate the activation of LINC00969, thereby inhibiting the transcription/post‐transcription of NLRP3, promoting the acquired gefitinib resistance, and suppressing the pyrodeath of lung cancer.^[^ ^26^ ^]^ Furthermore, Larsson et al. have uncovered that the H3K4me1 level falls to induce transcriptional dysregulation in multiple pathways, thus promoting colorectal cancer development.^[^ ^27^ ^]^

EBF2, a transcription factor with basic helix‐loop‐helix (bHLH) domain, participates in the differentiation and function of brown and beige adipocytes.^[^ ^28^, ^29^ ^]^ Stine et al. have reported that subcutaneous white adipose tissue or primary adipose cell cultures from EBF2 knockout mouse fail to trigger thermogenic programming in response to adrenergic stimulation.^[^ ^30^ ^]^ Conversely, EBF2 if its expression is restored in adipose tissues, can robustly stimulate beige adipocyte formation in the white adipose tissue of mice.^[^ ^30^ ^]^ Genome‐wide ChIP‐seq mapping and analysis have revealed that EBF2 preferentially moves to the enhancer regions of brown fat‐specific genes, thereby increasing RNA polymerase II and H3K27ac mark occupancies on brown fat‐selective cis elements.^[^ ^31^ ^]^ Having established that EBF2 regulates chromatin accessibility, Shapira et al. further uncovered that EBF2 cooperates with ATP‐dependent BAF (SWI/SNF) chromatin remodeling complexes to regulate chromatin structure.^[^ ^32^ ^]^ However, the roles of EBF2 and its associated molecules in PDAC pathogenesis remain unknown.

In this study, we identified EBF2 as an H3K4me1‐binding protein. Through RNA‐seq and ChIP‐seq analyses, we teased out KLLN as a direct target of KMT2D and EBF2 in PDAC cells, and further verified that KMT2D and EBF2 cooperatively inhibited the proliferation, migration, and invasion of PDAC cells through upregulating KLLN. These findings reveal a novel pathologic mechanism underlying PDAC progression and provide potential therapeutic targets for PDAC treatment.

## 2 Results

### 2.1 EBF2 is Identified as the H3K4me1‐Binding Protein

**Figure 1:**
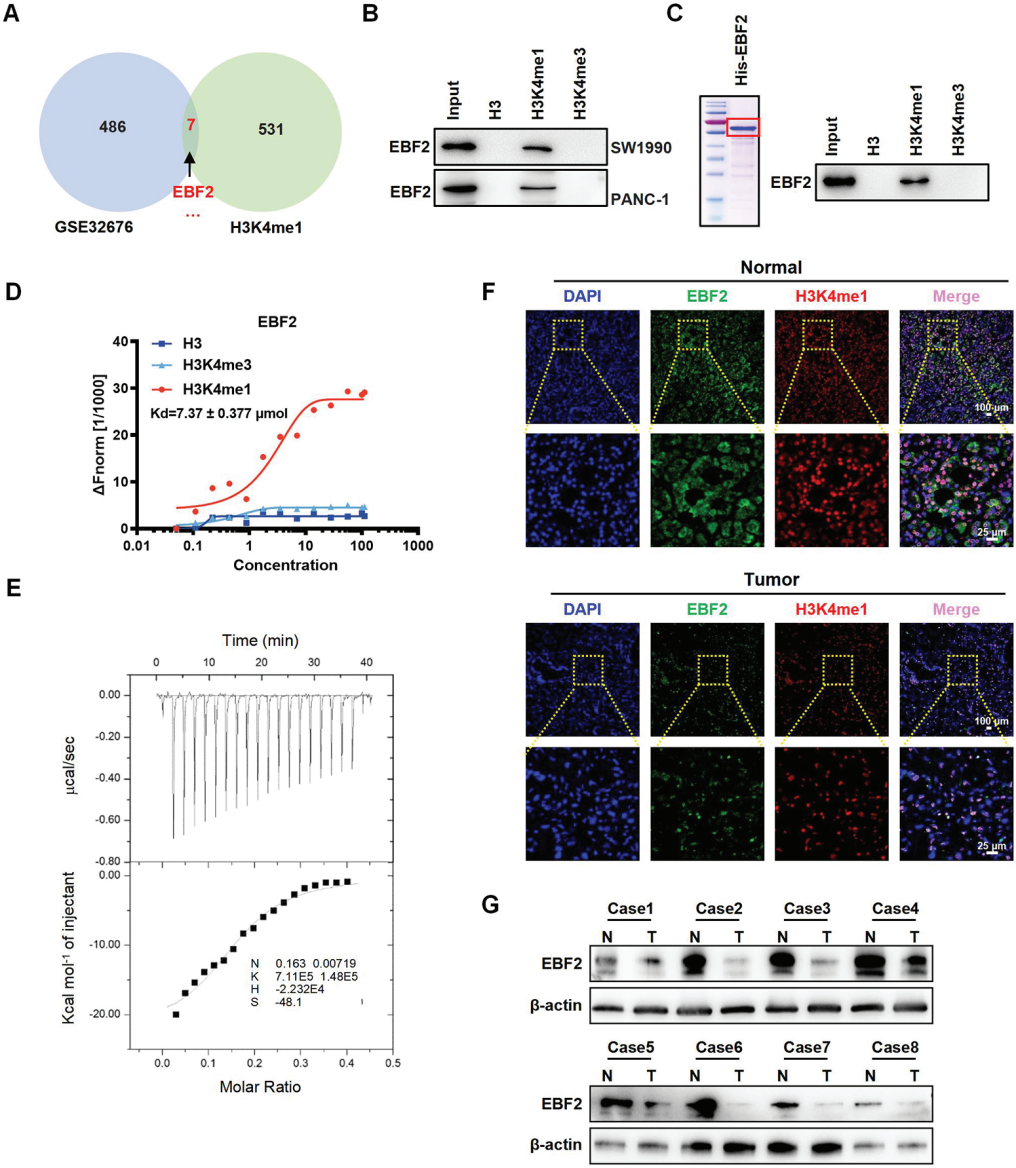
EBF2 is identified as an H3K4me1‐binding protein. A) Venn diagram showing the overlapping genes that were associated with H3K4me1 and significantly reduced in pancreatic cancer (GEO data, GSE32676). B) Peptide pull‐down assay to determine the interactions between H3, H3K4me1, or H3K4me3 peptides and EBF2 in vitro. C) Peptide pull‐down assay performed with purified His‐EBF2 fusion protein and H3, H3K4me1, or H3K4me3 peptides (right); Coomassie blue staining with SDS‐PAGE gel for the purified His‐EBF2 fusion protein expressed in *E. coli* (left). D) MST assay confirming the direct interactions between His‐EBF2 and H3, H3K4me1, or H3K4me3 peptides. Kd = 7.37 ± 0.377 µm. E) ITC binding curve of His‐EBF2 fusion protein with H3K4me1 peptide. Ka = 7.11 × 10^5^ ± 1.48 × 10^5^ mol^−1^. F) Immunofluorescence staining of EBF2 protein in PDAC and adjacent tissues. G) Western blot analysis for EBF2 protein level in PDAC and adjacent tissues.

Unmodified histone 3 (H3), H3K4me1 peptides were incubated with nuclear extract of SW1990 cells, separated by SDS‐PAGE gel, and subjected to mass spectrometry. Factors specifically associating with H3K4me1 were screened by peptide pulldown coupled with mass spectrometry analysis. Mass spectrometry‐based proteomic analysis yielded a plethora of putative H3K4me1‐associated proteins, including many known histone or DNA modifiers, readers, and chromatin remodelers (Table S1, Supporting Information). Given that KMT2D executes its antitumor function via catalyzing the formation of H3K4me1 in pancreatic cancer, we explored the genes associated with H3K4me1. Seven genes were identified in mass spectrometry and bioinformatic analysis, all significantly downregulated in pancreatic cancer (GEO data, GSE32676),^[^ ^33^ ^]^ including TSPYL2, TSR1, METAP2, CYR61, EBF2, CIRBP, and TGFBR3. Among them, EBF2, a transcription factor, had a helix‐loop‐helix structure (**Figure** **1**A). Using biotin‐labeled peptides and protein extracts from SW1990 and PANC‐1 cells, the pull‐down assay confirmed the preferential binding of EBF2 to H3K4me1 over H3K4me3 in vitro (Figure 1B).

The CDS region of EBF2 gene was inserted into pET‐28a plasmid to express recombinant His‐tagged EBF2 fusion protein (His‐EBF2) in *E. coli* (Figure 1C). We observed that purified His‐EBF2 was pulled down by H3K4me1 peptide, but not by H3K4me3 (Figure 1C). The MST assay suggested that His‐EBF2 directly bound to H3K4me1 peptide with a dissociation constant (Kd) of 7.37 ± 0.377 µm (Figure 1D). The ITC assay also showed a strong affinity of His‐EBF2 to H3K4me1 peptide (Figure 1E; Ka = 7.11 × 10^5^ ± 1.48 × 10^5^ mol^−1^), implying that EBF2 can specifically recognize H3K4me1. To gain insight into this recognition, we analyzed the crystal structure of human EBF2 in complex with a H3K4me1‐containing histone H3 tail peptide. Molecular docking showed that H3K4me1 nestled tightly in a pocket on the surface, which contained hydrophobic side chains of His239 (H239) of EBF2 (Figure S1A, Supporting Information). Co‐IP on SW1990 cells showed that the ability of EBF2‐H239A to bind H3K4me1 was significantly weaker than that of EBF2‐WT (Figure S1B, Supporting Information). This indicated that H239 is the site for H3K4me1 binding to EBF2 protein. We further examined the expression of EBF2 in PDAC and adjacent tissues. Immunofluorescence staining showed lower protein levels of EBF2 and H3K4me1 in PDAC tissues than in adjacent tissues (Figure 1F). Both EBF2 and H3K4me1 marks were co‐localized in the nuclei of PDAC or adjacent cells. Western blot analysis confirmed the reduction of EBF2 expression in PDAC tissues (Figure 1G), both in the nucleus and cytoplasm (Figure S1C, Supporting Information). These data demonstrated EBF2 as an H3K4me1‐associated protein, and the low expression of EBF2 in PDAC tissues supported that EBF2 may play a critical role in PDAC progression.

### 2.2 EBF2 Inhibits the Proliferation and Metastasis of PDAC Cells

**Figure 2:**
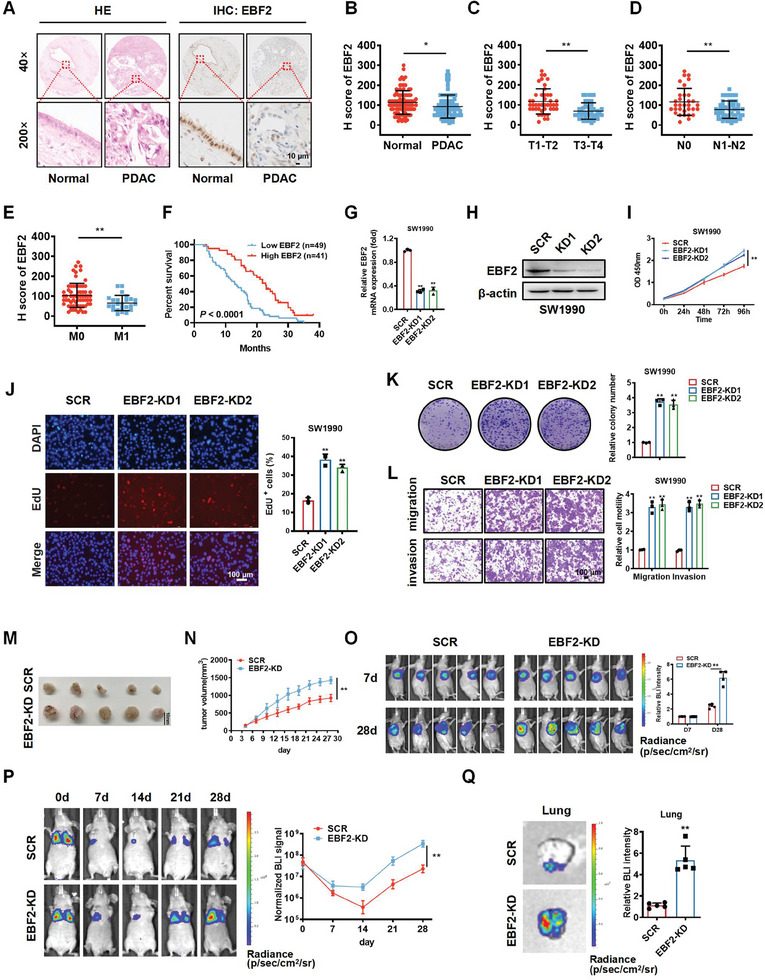
EBF2 is downregulated in PDAC tissues and inhibits cell proliferation and metastasis. A) H&E and IHC staining of EBF2 protein in PDAC and adjacent normal tissues. Scale bar, 10 µm. B) H scores of EBF2 in PDAC and adjacent normal tissues. C–E) Correlations of EBF2 expression with tumor stage (C), lymph node metastasis (D), and distal metastasis (E) in PDAC patients. Data in B–E are presented as mean ± SEM, ^*^ *p* < 0.05, ^**^ *p* < 0.01 by Student's *t*‐test. F) Kaplan–Meier plot of overall survival in 90 patients with PDAC, stratified by EBF2 expression (Log‐rank test, *p* < 0.0001). G, H) The mRNA (G) and protein (H) levels of EBF2 in SW1990 cells treated with EBF2 shRNAs (EBF2‐KD) or negative control (SCR) verified by qPCR and Western blot assays. Data in G are presented as mean ± SEM, ^**^ *p* < 0.01 by one‐way ANOVA test. I–L) CCK‐8 (I), EdU incorporation (J), colony‐formation (K) and Transwell (L) assays were performed to assess the effects of EBF2 knockdown on the proliferation, migration, and invasion of SW1990 cells in vitro. Data in I–L are presented as mean ± SEM, ^**^ *p* < 0.01 by two‐way ANOVA (I) and one‐way ANOVA (J–L) test. M) Images of subcutaneous tumor xenografts in SCR and EBF2‐KD mice. N) The tumor growth curves of xenografts were plotted in SCR and EBF2‐KD mice. O) BALB/c nude mice with subcutaneous tumor xenografts in SCR and EBF2‐KD group were imaged with in vivo imaging system at different time points. P) BALB/c nude mice injected with SCR and EBF2‐KD SW1990 cells via tail vein were imaged by in vivo imaging system at different time points. Data in N–P are presented as mean ± SEM, ^**^ *p* < 0.01 by two‐way ANOVA test (n = 5). Q) Representative images of lung metastasis loci in SCR and EBF2‐KD mice. Data in Q are presented as mean ± SEM, ^**^ *p* < 0.01 by Student's *t*‐test (n = 5).

To evaluate the expression of EBF2 in PDAC tissues, a tissue microarray (TMA) was constructed, which comprised 90 pairs of PDAC and corresponding adjacent normal tissue samples. The TMA was subjected to IHC staining, showing that EBF2 was mainly located in the nuclei of both normal and PDAC tumor cells (**Figure** **2**A). H scores revealed that the protein level of EBF2 was significantly downregulated in PDAC tissues as compared to that in the normal tissues (Figure 2B). The expression level of EBF2 was negatively correlated with tumor stage, lymph node metastasis, and distal metastasis in PDAC patients (Figure 2C–E). Kaplan–Meier survival curves showed that a lower EBF2 expression was significantly associated with a worse survival (Figure 2F).

To evaluate the effect of EBF2 on the biological behavior of PDAC cells, SW1990 and PANC‐1 cell lines stably expressing EBF2 shRNA were obtained by puromycin screening. EBF2 depletion in SW1990 and PANC‐1 cells were confirmed by qPCR and Western blot (Figure 2G,H; Figure S1D,E, Supporting Information). CCK‐8, EdU incorporation, colony‐formation, and Transwell assays were applied to assess the effects of EBF2 on cell proliferation, migration, and invasion of PDAC cells in vitro. EBF2‐depleted SW1990 and PANC‐1 cells exhibited more pronounced proliferation, migration, and invasion than control cells (Figure 2I–L; Figure S1F–I, Supporting Information). Furthermore, silencing EBF2 significantly facilitated the growth of SW1990‐luc xenografts in vivo (Figure 2M–O). To examine the metastatic behavior of SW1990 cells in vivo, EBF2‐depleted SW1990‐luc cells were injected into the tail veins of BALB/c mice. Through live‐imaging observations, we found that EBF2 deficiency promoted the formation of metastatic foci in the lungs of mice (Figure 2P,Q). H&E staining showed that both liver and lung metastases increased in mice injected with EBF2‐depleted SW1990‐luc cells (Figure S1J,K, Supporting Information). These data were consistent with the association of EBF2 downregulation with PDAC progression.

### 2.3 KMT2D Expression is Positively Correlated with EBF2 Expression in PDAC Tissues and KMT2D Depletion Induces the Phenotype of EBF2 Knockout

**Figure 3:**
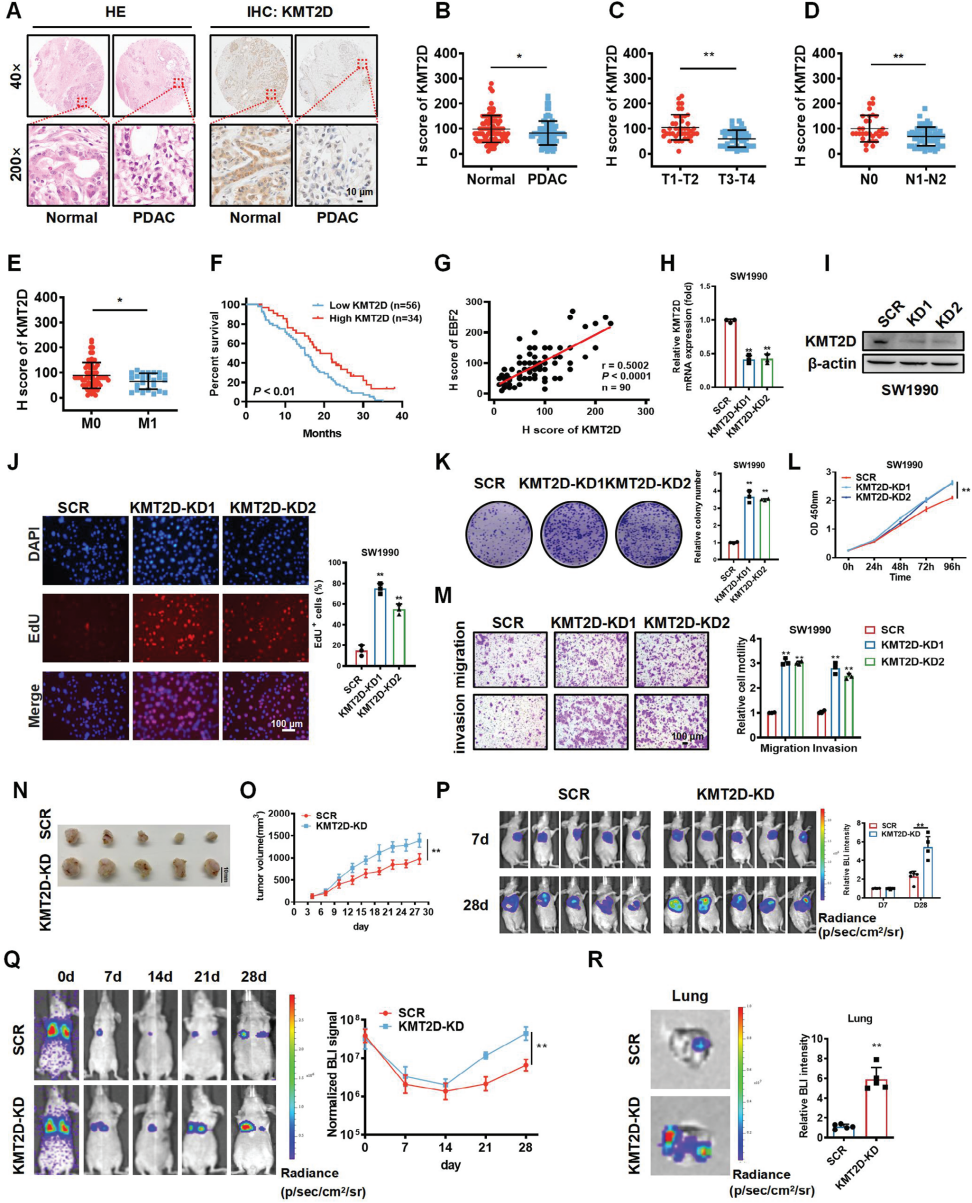
KMT2D expression is positively correlated with EBF2 expression in PDAC tissues and copies the phenotype caused by EBF2. A) H&E and IHC staining of KMT2D protein in PDAC and adjacent normal tissues. Scale bar, 10 µm. B) H score of KMT2D in PDAC and adjacent normal tissues. C–E) Correlations of KMT2D expression with tumor stage (C), lymph node metastasis (D), and distal metastasis (E) of PDAC patients. Data in B–E are presented as mean ± SEM, ^*^ *p* < 0.05, ^**^ *p* < 0.01 by Student's *t*‐test. F) Kaplan–Meier plot of overall survival in 90 patients with PDAC, stratified by KMT2D expression (Log‐rank test, *p* < 0.01). G) Spearman correlation analysis of KMT2D and EBF2 levels in PDAC tissues (n = 90, r = 0.5002, *p* < 0.0001). H, I) The mRNA (H) and protein (I) levels of KMT2D in SW1990 cells verified by qPCR and Western blot assays. Data in H are presented as mean ± SEM, ^**^ *p* < 0.01 by one‐way ANOVA test. J–M) EdU incorporation (J), colony‐formation (K), CCK‐8 (L), and Transwell (M) assays assessed the effects of KMT2D knockdown on the proliferation, migration, and invasion of SW1990 cells in vitro. Data in J–M are presented as mean ± SEM, ^**^ *p* < 0.01 by two‐way ANOVA (L) and one‐way ANOVA (J, K, and M) test. N) Images of subcutaneous tumor xenografts in KMT2D knockdown (KMT2D‐KD) and control (SCR) mice. O) The tumor growth curves of xenografts were plotted in SCR and KMT2D‐KD mice. P) BALB/c nude mice with subcutaneous tumor xenografts in SCR and EBF2‐KD group were imaged with in vivo imaging system at different time points. Q) BALB/c nude mice injected with SCR and EBF2‐KD SW1990 cells through the tail vein were imaged by in vivo imaging system at different time points. Data in O–Q are presented as mean ± SEM, ^**^ *p* < 0.01 by two‐way ANOVA test (n = 5). R) Representative images of lung metastasis loci in SCR and KMT2D‐KD mice. Data in R are presented as mean ± SEM, ^**^ *p* < 0.01 by Student's *t*‐test (n = 5).

KMT2D, also known as ALR/MLL4, catalyzes the formation of H3K4me1 on promoters or enhancers to regulate cell‐type‐specific gene expression and cell fate transition.^[^ ^18^, ^22^, ^24^ ^]^ However, the clinical significance and biological roles of KMT2D in PDAC remain to be clarified. Through IHC staining, we found that KMT2D was significantly downregulated in PDAC tissues (**Figure** **3**A,B), and negatively correlated with tumor stage, lymph node metastasis, and distal metastasis in PDAC patients (Figure 3C–E). Kaplan–Meier survival curves show that KMT2D downregulation predicted a poor survival (Figure 3F). Subsequent Spearman correlation analysis demonstrated that KMT2D expression was positively correlated with EBF2 expression in PDAC tissues (n = 90, r = 0.5002, *p* < 0.0001) (Figure 3G).

We established SW1990 and PANC‐1 cell lines with stable KMT2D knockdown to evaluate the role of KMT2D in PDAC (Figure 3H,I; Figure S2A,B, Supporting Information). CCK‐8, EdU incorporation, colony‐formation, and Transwell assays uncovered that knockdown of KMT2D significantly enhanced the proliferation, migration, and invasion of SW1990‐luc and PANC‐1 cells (Figure 3J–M; Figure S2C–F, Supporting Information), which mimics the phenotype caused by EBF2 depletion. In vivo results demonstrated that knockdown of KMT2D significantly promoted the growth of SW1990‐luc xenografts (Figure 3N–P), as well as the formation of metastatic foci in mouse lungs (Figure 3Q,R; Figure S2G,H, Supporting Information). These results revealed that KMT2D could attenuate PDAC growth and metastasis.

### 2.4 KLLN is a Common Transcriptional Target of KMT2D and EBF2 in PDAC Cells

**Figure 4:**
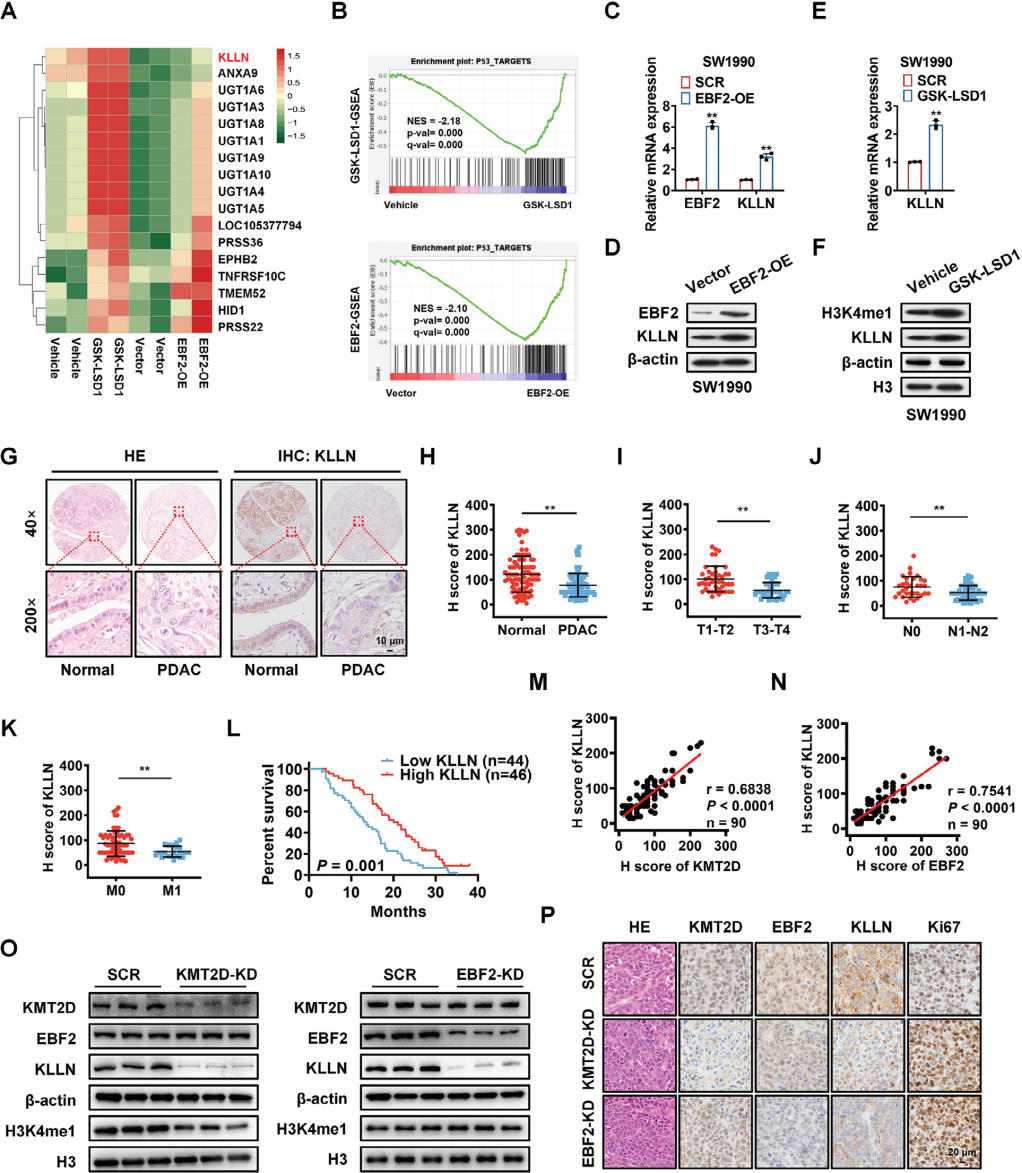
KLLN is a common transcriptional target of KMT2D and EBF2 in PDAC cells. A) Heatmaps from RNA‐seq data showing overlapping of differentially expressed genes (DEGs) of SW1990‐luc cells with EBF2 overexpression (EBF2‐OE) or H3K4me1 activation by GSK‐LSD1. B) Gene Set Enrichment Analysis (GSEA) reveals that the DEGs are enriched in genes regulated by p53. C, D) The mRNA (C) and protein (D) levels of EBF2 and KLLN in negative control (Vector) and EBF2 overexpressed (EBF2‐OE) SW1990 cells verified by qPCR and Western blot analysis. Data in C are presented as mean ± SEM, ^**^ *p* < 0.01 by Student's *t*‐test. E,F) The mRNA (E) and protein (F) levels of KLLN in negative control (Vector) and GSK‐LSD1‐treated SW1990 cells verified by qPCR and Western blot analysis. Data in E are presented as mean ± SEM, ^**^ *p* < 0.01 by Student's *t*‐test. G) H&E and IHC staining of KLLN protein in PDAC and adjacent normal tissues. Scale bar, 10 µm. H) H score of KLLN in PDAC and tumor adjacent tissues. I–K) Correlations of KLLN expression with tumor stage (I), lymph node metastasis (J), and distal metastasis (K) in PDAC patients. Data in I–K are presented as mean ± SEM, ^**^ *p* < 0.01 by Student's *t*‐test. L) Kaplan–Meier plot of overall survival of 90 patients with PDAC, stratified by KLLN expression (Log‐rank test, *p* = 0.001). M) Spearman correlation analysis of KMT2D and KLLN levels in PDAC tissues (n = 90, r = 0.6838, *p* < 0.0001). N) Spearman correlation analysis of EBF2 and KLLN levels in PDAC tissues (n = 90, r = 0.7541, *p* < 0.0001). O) Western blot analysis of KLLN and H3K4me1 levels in SCR, KMT2D‐KD, or EBF2‐KD xenografts. P) H&E and IHC staining of KLLN and Ki67 in SCR, KMT2D‐KD, or EBF2‐KD xenografts. Scale bar, 20 µm.

Next, we sought to elucidate the roles of KMT2D‐ and EBF2‐regulated H3K4me1 through profiling the gene transcription in PDAC cells with H3K4me1 activation by GSK‐LSD1 or EBF2 overexpression (EBF2‐OE). GSK‐LSD1, as a selective inhibitor of KDM1A/LSD1, can increase H3K4me1 level via blocking the histone H3K4 demethylase activity of KDM1A/LSD1.^[^ ^34^, ^35^, ^36^, ^37^ ^]^ RNA‐seq revealed that the upregulation of EBF2 or H3K4me1 causes transcriptional alterations in SW1990 cells. These differentially expressed genes (DEGs) were overlapped genes between EBF2‐OE and GSK‐LSD1‐treated SW1990 cells. A total of 17 upregulated genes were screened by RNA‐seq (**Figure** **4**A). Among them, we selected KLLN for further investigation, for that it has been identified as a p53‐dependent tumor suppressor or an inducer of S and G2 phase checkpoint control.^[^ ^38^, ^39^ ^]^ Gene Set Enrichment Analysis (GSEA) revealed that these DEGs were enriched for genes regulated by p53 (Figure 4B). The qPCR and Western blot assays validated the significant upregulation of KLLN mRNA and protein levels in EBF2‐OE and GSK‐LSD1‐treated SW1990 cells (Figure 4C–F). Moreover, qPCR and Western blot assays verified that the mRNA and protein levels of KLLN significantly downregulated in KMT2D‐ or EBF2‐depleted SW1990 and PANC‐1 cells (Figure S3A–D, Supporting Information). ChIP‐qPCR assay was further performed to examine the binding of KMT2D, H3K4me1, and EBF2 at the promoter of KLLN gene in SW1990 cells. A series of oligonucleotide primers were designed for sequencing the proximal promoter region of the KLLN gene (−1831 to TSS). ChIP‐qPCR results revealed significant KMT2D, H3K4me1, and EBF2 signals in the promoter region of KLLN gene, especially the −500 bp upstream region (Figure S3E, Supporting Information). EBF2 knockdown (EBF2‐KD) markedly blocked the binding of EBF2, but not H3K4me1 in SW1990 cells (Figure S3F, Supporting Information). Interestingly, KMT2D knockdown (KMT2D‐KD) also significantly inhibited the bindings of KMT2D, H3K4me1, and EBF2 in SW1990 cells (Figure S3G, Supporting Information).

To further explore the genome‐wide occupancy of KMT2D‐dependent H3K4me1 and EBF2, we performed ChIP‐seq and CUT&Tag analyses using SW1990 cells. ChIP‐seq revealed a prominent reduction in KMT2D, H3K4me1, and EBF2 signals at the transcription start site (TSS) and promoter regions of genes in KMT2D‐depleted (KMT2D‐KD) cells (Figure S3H–J, Supporting Information). These results were validated by the CUT&Tag data (Figure S3K–M, Supporting Information). IHC staining exhibited that KLLN was also significantly downregulated in PDAC tissues (Figure 4G,H), and its expression was negatively correlated with tumor stage, lymph node metastasis, and distal metastasis in PDAC patients (Figure 4I–K). Kaplan–Meier survival curves showed that patients with a low expression of KLLN exhibited a poor survival (Figure 4L). Spearman correlation analysis (Figure 4M,N) showed that KLLN expression was positively correlated with KMT2D (n = 90, r = 0.6838, *p* < 0.0001) or EBF2 expression in PDAC tissues (n = 90, r = 0.7541, *p* < 0.0001). Western blot analysis and IHC staining further verified that KLLN downregulation in KMT2D‐ or EBF2‐depleted xenograft tissues was accompanied by increased Ki67 expression (Figure 4O,P). These data indicated that KLLN was regulated by both KMT2D and EBF2.

### 2.5 GSK‐LSD1 Inhibits the Proliferation, Migration and Invasion of PDAC Cells

**Figure 5:**
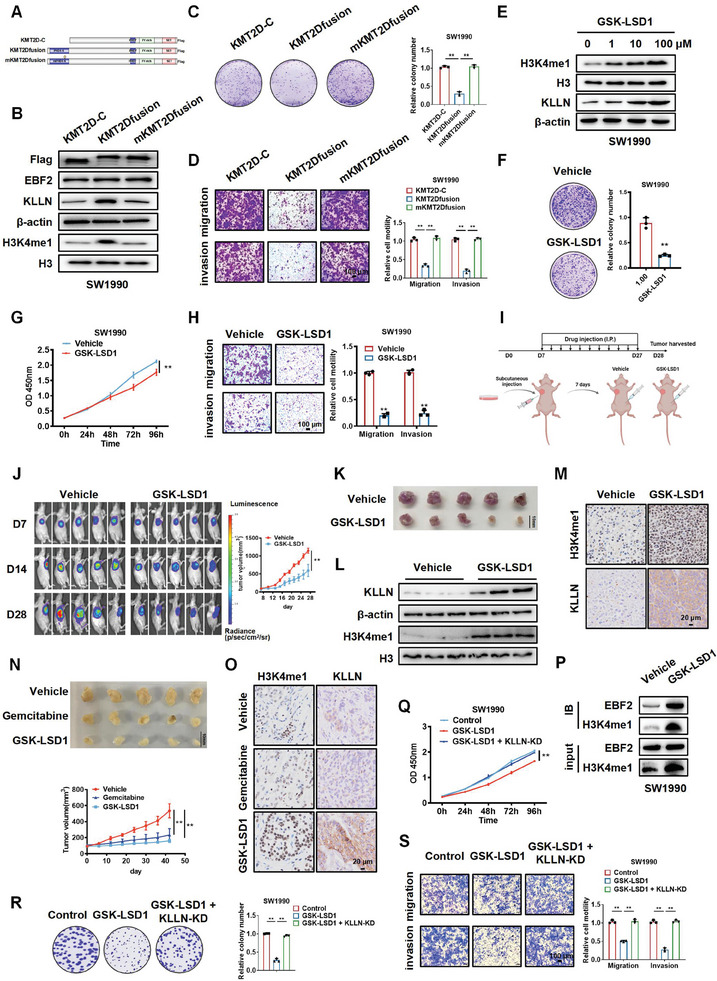
GSK‐LSD1 inhibits the proliferation, migration, and invasion of PDAC cells. A) Diagram of flag‐tagged KMT2D‐C, KMT2Dfusion and mKMT2Dfusion plasmid models. B) Western blot analysis of KLLN and H3K4me1 levels in SW1990 cells transfected with KMT2D‐C, KMT2D_fusion,_ and mKMT2D_fusion_. C, D) Colony‐formation (C) and Transwell (D) assays of SW1990 cells transfected with KMT2D‐C, KMT2D_fusion,_ and mKMT2D_fusion_. Data in C and D are presented as mean ± SEM, ^**^ *p* < 0.01 by one‐way ANOVA test. E) Western blot analysis of H3K4me1 and KLLN levels in SW1990 cells treated with 0, 1, 10, or 100 µm GSK‐LSD1. F–H) Colony‐formation (F), CCK‐8 (G), and Transwell (H) assays of SW1990 cells treated with Vehicle and GSK‐LSD1. Data in F–H are presented as mean ± SEM, ^**^ *p* < 0.01 by Student's *t*‐test (F and H), two‐way ANOVA test (G). I, J) BALB/c nude mice injected with SW1990‐luc cells were treated with Vehicle or GSK‐LSD1, and imaged at different time points by in vivo imaging system. Data in J are presented as mean ± SEM, ^**^ *p* < 0.01 by two‐way ANOVA test (n = 5). K) Images of subcutaneous tumor xenografts in Vehicle and GSK‐LSD1 mice. L,M) Western blot (L) and IHC staining (M) analysis of KLLN and H3K4me1 levels in control or GSK‐LSD1‐treated lung metastasis tissues. N) Images of patient‐derived xenograft (PDX) models treated with vehicle, gemcitabine, or GSK‐LSD1. Data in N are presented as mean ± SEM, ^*^ *p* < 0.05, ^**^ *p* < 0.01 by two‐way ANOVA test (n = 5). O) IHC staining analysis of KLLN and H3K4me1 levels in PDX tumors treated with vehicle, gemcitabine, or GSK‐LSD1. P) Co‐IP assay of proteins immunoprecipitated with H3K4me1 antibodies from lysates of SW1990 cells treated with vehicle control and GSK‐LSD1. Q–S) CCK‐8 (Q), colony‐formation (R) and Transwell (S) assays were used to detect the proliferation, migration, and invasion of control, GSK‐LSD1, and GSK‐LSD1+KLLN SW1990 cells. Data in Q–S are presented as mean ± SEM, ^**^ *p* < 0.01 by two‐way ANOVA test (Q) and one‐way ANOVA test (R and S).

To evaluate the performance of KMT2D‐mediated H3K4me1 in the proliferation, migration, and invasion of PDAC cells, we constructed KMT2D‐C, KMT2D_fusion_, and methyltransferase activity‐deficient mutant KMT2D_fusion_ C1523A (mKMT2D_fusion_) plasmids, as previously reported (**Figure** **5**A).^[^ ^40^ ^]^ We then transfected these plasmids into SW1990 and PANC‐1 cells, finding that KMT2D_fusion_ overexpression activated the expression of H3K4me1 and KLLN, while mKMT2D_fusion_ did not (Figure 5B; Figure S4A, Supporting Information). Colony formation and Transwell assays showed that KMT2D_fusion_ significantly impeded the proliferation, migration, and invasion of SW1990 and PANC‐1 cells in vitro (Figure 5C,D; Figure S4B,C, Supporting Information).

Western blot analysis showed that GSK‐LSD1 increased the levels of H3K4me1 and KLLN in a concentration‐dependent manner (Figure 5E; Figure S4D, Supporting Information), and significantly inhibited the proliferation, migration, and invasion of SW1990 and PANC‐1 cells (Figure 5F–H; Figure S4E–G, Supporting Information). Consistently, in vivo experiments showed that GSK‐LSD1‐treated mice presented significantly smaller sizes of SW1990‐luc xenografts compared to control mice (Figure 5I–K). The upregulation of H3K4me1 and KLLN in these metastatic foci was verified by Western blot and IHC analyses (Figure 5L,M). Subsequently, a patient‐derived xenograft (PDX) model of PDAC was successfully established, and the tumor growth was monitored under different treatments. Compared with control mice, GSK‐LSD1‐treated mice developed remarkably smaller tumors in which the expression of H3K4me1 was significantly upregulated. Notably, GSK‐LSD1 therapy exhibited more favorable effects than Gemcitabine, which was a commonly used medication for PDAC patients (Figure 5N,O). To prove the linkage between EBF2 and H3K4me1, we performed Co‐IP in SW1990 cells. As shown in Figure 5P, GSK‐LSD1 treatment increased H3K4me1 level without affect EBF2 expression, and co‐immunoprecipitated more EBF2, which may be due to the upregulation of H3K4me1. To explore whether GSK‐LSD1 fulfills its tumor‐suppressing role by upregulating KLLN, rescue experiments were performed. As shown in Figure 5Q–S and Figure S4H–J (Supporting Information), knockdown of KLLN markedly attenuated the tumor‐suppressive phenotype of GSK‐LSD1 in SW1990 and PANC‐1 cells.

### 2.6 KMT2D and EBF2 Cooperate to Activate the Expression of KLLN

**Figure 6:**
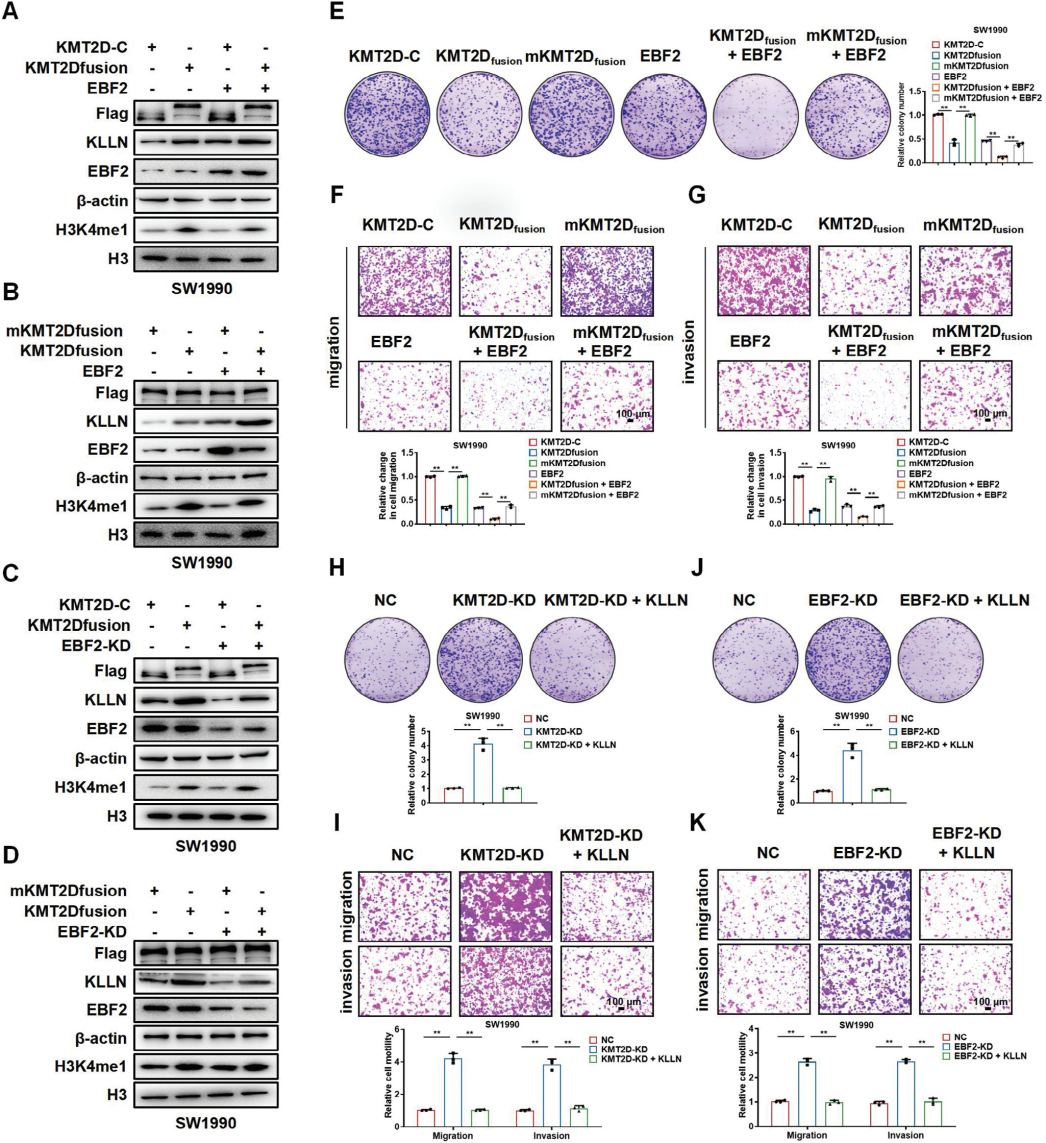
KMT2D and EBF2 cooperate to regulate the expression of KLLN. A) Western blot of KLLN and H3K4me1 in SW1990 cells transfected with KMT2D‐C, KMT2D_fusion_, or EBF2. B) Western blot of KLLN and H3K4me1 in SW1990 cells transfected with mKMT2D_fusion_, KMT2D_fusion_ or EBF2. C) Western blot of KLLN and H3K4me1 in SW1990 cells transfected with KMT2D‐C, KMT2D_fusion_ or EBF2‐KD. D) Western blot of KLLN and H3K4me1 in SW1990 cells transfected with mKMT2D_fusion_, KMT2D_fusion_, or EBF2‐KD. β‐actin and H3 were used as loading control. E–G) Colony‐formation (E), migration (F), and invasion (G) of SW1990 cells treated with KMT2D‐C, KMT2D_fusion_, mKMT2D_fusion_, EBF2, KMT2D_fusion_+EBF2, mKMT2D_fusion_+EBF2. H,I) Colony‐formation assays (H) and Transwell (I) assays of SW1990 cells transfected with control (NC), KMT2D‐KD or KMT2D‐KD + KLLN. J‐K) Colony‐formation assays (J) and Transwell (K) assays of SW1990 cells transfected with control (NC), EBF2‐KD or EBF2‐KD+KLLN. Scale bar,100 µm. Data in E–K are presented as mean ± SEM, ^**^ *p* < 0.01 by one‐way ANOVA test.

To gain insight into the molecular mechanisms by which KMT2D and EBF2 regulate KLLN, we manipulated the expression of KMT2D or EBF2 in SW1990 and PANC‐1 cells, and determined the expression of KLLN by Western blot. Interestingly, the cells co‐expressing KMT2D_fusion_ and EBF2 exhibited a greater KLLN activity than those transfected with KMT2D_fusion_ or EBF2 alone, suggesting KMT2D and EBF2 might cooperate to activate the expression of KLLN in SW1990 (**Figure** **6**A,B) and PANC‐1 cells (Figure S5A,B, Supporting Information). When EBF2 was knocked down (EBF2‐KD), KMT2D_fusion_ imposed a weaker effect on the expression of KLLN in SW1990 (Figure 6C,D) and PANC‐1 cells (Figure S5C,D, Supporting Information), indicating that EBF2 mediated this cooperative effect.

To determine the effect of KMT2D and EBF2 on cell phenotype, we manipulated the expression of KMT2D or EBF2 in SW1990 and PANC‐1 cells and assessed their proliferation, migration, and invasion in vitro. Compared with KMT2D‐C or mKMT2D_fusion_ control, KMT2D_fusion_ overexpression significantly inhibited the proliferation, migration, and invasion of SW1990 and PANC‐1 cells. Moreover, the cells co‐expressing KMT2D_fusion_ and EBF2 exhibited a greater inhibitory effect on the proliferation, migration, and invasion of SW1990 and PANC‐1 cells than those transfected with KMT2D_fusion_ or EBF2 alone (Figure 6E–G; Figure S5E–G, Supporting Information).

Rescue experiments were also performed to testify whether the tumor suppressive effect of KMT2D and EBF2 is mediated by KLLN. As shown in Figure 6H–K and Figure S5H–K (Supporting Information), knockdown of KMT2D or EBF2 enhanced the proliferation, migration, and invasion of SW1990 and PANC‐1 cells. Interestingly, rescuing KLLN expression significantly reversed the phenotypic changes of SW1990 and PANC‐1 cells induced by KMT2D or EBF2 knockdown. Taken together, KMT2D and EBF2 might inhibit the proliferation, migration, and invasion of PDAC cells through targeting KLLN. Colony formation and Transwell assays indicated that EBF2 overexpression significantly abolished the promotive effects of KMT2D on the proliferation, migration, and invasion of SW1990 cells, but knockdown of KMT2D plus EBF2, compared to KMT2D or EBF2 knockdown alone, enhanced those processes of SW1990 cells (Figure S6A–D, Supporting Information). Taken together, KMT2D and EBF2 inhibited the proliferation, migration, and invasion of PDAC cells through targeting KLLN.

## 3 Discussion

In the present study, we reveal that H3K4me1 is recognized by EBF2, a transcription factor with a helix‐loop‐helix structure. Our results demonstrate that, through interacting with KMT2D‐dependent H3K4me1 and epigenetically upregulating KLLN (also known as killin), EBF2 inhibits PDAC cell proliferation, migration, and invasion.

H3K4me1 is an evolutionarily conserved histone modification, catalyzed by the COMPASS‐like methyltransferase family, including KMT2C and KMT2D.^[^ ^22^, ^23^, ^24^ ^]^ H3K4me1 demarcates the boundaries of active enhancers or promoters, thus limiting the recruitment of effectors or modulators.^[^ ^41^ ^]^ Therefore, factors that can specifically recognize H3K4me1 would facilitate histone modification in epigenetic regulation. Previous studies in *Arabidopsis* have reported that the CW domain of histone methyltransferase SDG8, a zinc‐binding domain with conserved cysteines and tryptophans, was responsible for binding H3K4me1.^[^ ^42^, ^43^ ^]^ However, a crystal structure analysis has revealed that the hydrophobic, narrow pocket of the CW domain of SDG8 bring about steric hindrance that prohibits the binding of highly methylated lysine.^[^ ^43^ ^]^ In line with this, the CW domains in mammalian MORC1, MORC2, and LSD2 proteins are unable to recognize any methylated H3K4 peptides, whereas those of mammalian ZCWPW1, ZCWPW2, MORC3, and MORC4 can bind to unmethylated or tri‐methylated H3K4.^[^ ^44^, ^45^, ^46^ ^]^ Local et al. have revealed that the PHD2 domain of Double PHD fingers 3 (DPF3) enables the preferential recognition of H3K4me1 over H3K4me3, thereby facilitating the recruitment of BRG1‐associated factor (BAF) chromatin remodeling complex to enhancers in mammalian cells.^[^ ^47^ ^]^ Through mass spectrometry‐based proteomic analysis and in vitro peptide/protein interaction assays, we here identified that transcription factor EBF2 can pinpoint H3K4me1, and distinguish it from H3K4me3. A previous study has shown that EBF2 physically acts on the chromatin remodeler Brahma‐Related Gene 1 (BRG1) and the BAF chromatin remodeling complex in brown adipocytes.^[^ ^32^ ^]^ However, ChIP analysis revealed that EBF2 binding to brown fat genes, such as UCP1, was not interrupted by DPF3 or BRG1 deficiency, suggesting that EBF2 binding precedes BAF‐mediated chromatin remodeling. Notably, structural and functional analyses uncovered that EBF1, another member of the EBF family, could recognize nucleosome‐enriched DNA to increase chromatin accessibility through its C‐terminal domain.^[^ ^48^, ^49^ ^]^ Taken together, EBF proteins can directly contact with chromatin in gene regulation. Although the DNA‐binding domain of EBF2 shares 92% of its amino acid sequences with EBF1,^[^ ^50^, ^51^ ^]^ future studies are required to explore whether EBF2 directly binds to nucleosomal DNA or histone modifications, particularly H3K4me1.

KMT2D, also known as ALR/MLL4, catalyzes the formation of H3K4me1 on promoters or enhancers of cell‐type‐specific genes to regulate gene expression and cell fate transition.^[^ ^18^, ^40^, ^41^ ^]^ Multiple studies have dug into the roles of KMT2D in the initiation and progression of various cancers. For example, loss of KMT2D in the early stage can collaborate with the upregulation of Bcl‐2 oncogene to facilitate lymphoma development.^[^ ^52^, ^53^ ^]^ Lung‐specific KMT2D loss significantly promotes lung tumorigenesis and pro‐tumorigenic programming in mice, including glycolysis, supporting its role as a tumor suppressor.^[^ ^54^ ^]^ In line with these studies, the function analysis in the present study uncovered that knockdown of KMT2D dramatically boosted the proliferation, migration, and invasion of PDAC cells. However, recent studies have also suggested that KMT2D greatly enhanced the proliferation, invasion, and tumor formation of gastric cancer or prostate cancer cells,^[^ ^55^, ^56^ ^]^ implying that KMT2D acts on the tumor in either a promotive or a suppressive manner. Given that KMT2D catalyzes the formation of H3K4me1 on cell‐type‐specific gene enhancers or promoters, we hypothesized that its function is largely dependent on the H3K4me1‐binding factors. This hypothesis was supported by our function analysis that knockdown of KMT2D copied the phenotype caused by EBF2 in vitro and in vivo.

KLLN is located in 10q23.31 and originally identified as a target of p53 involved in the S‐phase regulation.^[^ ^38^ ^]^ Bennett et al. have shown that the KLLN promoter is hypermethylated in patients with Cowden syndrome without germline mutations in PTEN.^[^ ^57^ ^]^ As the first predisposition gene for such syndrome, KLLN confers a high risk of breast, thyroid, and other cancers.^[^ ^57^ ^]^ KLLN downregulation is significantly associated with high Gleason scores, indicating KLLN as a diagnostic or prognostic biomarker for advanced prostate carcinomas.^[^ ^58^ ^]^ In breast cancer cells, Sankunny et al. have demonstrated that KLLN mediated DNA damage‐induced apoptosis through the pathway of p53 phosphorylation and acetylation.^[^ ^59^ ^]^ Wang et al. have reported that transcription factor KLLN induces cell cycle arrest and apoptosis in breast cancer cells by directly promoting the transcription of TP53 and TP73.^[^ ^60^ ^]^ KLLN may act as a tumor suppressor and regulates cell cycle and apoptosis in diverse cancers. In the present study, we found that KLLN was downregulated in PDAC tissues, and rescuing its expression largely reversed the phenotype changes caused by EBF2 or KMT2D knockdown. These findings suggest that KLLN serves as a critical effector downstream KMT2D and EBF2 in the regulation of PDAC progression. Nevertheless, we could not rule out the possibility that KMT2D and EBF2 may target other genes to regulate PDAC progression. There are still limitations in this study. In particular, the enrichment of EBF2 and H3K4me1 in the KLLN promoter region was not very significant in the ChIP‐seq and CUT&Tag data. This may due to the low expression of KMT2D, EBF2, and H3K4me1 in PDAC cells or the decrease of measurement sensitivity caused by random errors or interference. These factors can thereby culminate an unstable targeting outcome for individual gene. For defining the regulation of KMT2D, EBF2, and H3K4me1 in PDAC, the future studies are required to validate and characterize the enrichment of EBF2 and H3K4me1 in the KLLN promoter region by using other highly sensitive novel technologies.

**Figure 7:**
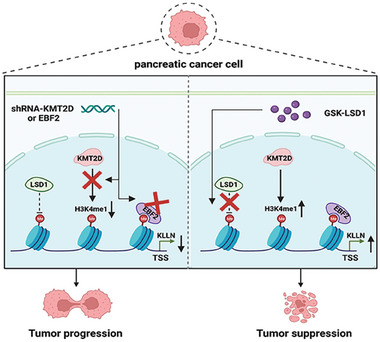
A diagram of EBF2 interacting with KMT2D‐mediated H3K4me1 to suppress pancreatic cancer progression.

In conclusion, we screened out EBF2 as a novel H3K4me1‐binding protein. EBF2 and KMT2D work together to regulate H3K4me1, posing strong activation on KLLN and inhibition on PDAC progression (**Figure** **7**). The interplay between H3K4me1 histone modification and KLLN transcription could be exploited to design novel therapeutic strategies for PDAC.

### 3.1 Abbreviations

KMT2D: Histone‐lysine N‐methyltransferase 2D; H3K4me1: Monomethylation of histone H3 at Lysine 4; EBF2: Early B‐cell factor 2;KLLN: Killin, p53 regulated DNA replication inhibitor; PDAC: Pancreatic cancer; TSS: transcription start site; LSD1: lysine‐specific demethylase 1; KMTs: histone lysine methyltransferases; KDMs: histone lysine demethylases; shRNA: Short hairpin RNA; siRNA: Small interfering RNA; H&E: Hematoxylin and eosin; SDS‐page; sodium dodecyl sulfate‐polyacrylamide gel electrophoresis; IPTG: isopropyl‐β‐D‐thiogalactoside; MST: Microscale thermophoresis; ITC: Isothermal titration calorimetry; RNA‐seq: qPCR: Quantitative real‐time PCR; RNA sequencing; CCK‐8: Cell Counting Kit‐8; EdU: 5‐Ethynyl‐2′‐deoxyuridine; ChIP: Chromatin immunoprecipitation; ChIP‐seq: Chromatin immunoprecipitation sequencing; Cleavage Under Targets and Tagmentation (CUT&Tag); IHC immunohistochemical; PDX: Patient‐derived xenograft; DEGs: Differentially expressed genes; GSEA: Gene Set Enrichment Analysis; Brg1: Brahma‐related gene 1; BAF: BRG1‐associated factor; DPF3: Double PHD fingers 3.

## 4 Experimental Section

### Cell Culture

Human pancreatic cancer cell lines SW1990 and PANC‐1 were purchased from the American Type Culture Collection (ATCC, USA), and cultured in Dulbecco's modified Eagle's medium (DMEM; Gibco, USA) or Roswell Park Memorial Institute 1640 medium (RPMI‐1640; Gibco) supplemented with 10% fetal calf serum (FCS; Wisent, Canada) and penicillin‐streptomycin solution (100 µg mL^−1^; Beyotime, China) in a humidified chamber with 5% CO_2_ at 37 °C.

### Cell Transfection and siRNA Interference Assays

SiRNAs against KMT2D and EBF2 were synthesized by GenePharma (Shanghai, China). SW1990 and PANC‐1 cells were transfected with oligonucleotides or indicated plasmids using Lipofectamine 2000 (Invitrogen, USA) according to the manufacturer's instructions. The sequences of siRNAs included KMT2D siRNA‐1: 5′‐GAGUCGAACUUUACUGUCUCC‐3′; KMT2D siRNA‐2: 5′‐CCACUCUCAUCAAAUCCGACA‐3′. EBF2 siRNA‐1: 5′‐GAGGUGACAUUAUCUUAUA‐3′; EBF2 siRNA‐2: 5′‐GCACUCACUACAAGUUACA‐3′.

### Mass Spectrometry

C‐terminal biotin‐tagged 19 amino acid N‐terminal peptides of H3, H3K4me1, and H3K4me3 were incubated with nuclear extract of SW1990 cells, and then with high‐capacity streptavidin agarose (Thermo Scientific, USA) for immunoprecipitation. The product was separated by sodium dodecyl sulfate‐polyacrylamide gel electrophoresis (SDS‐PAGE) with silver staining. Protein bands of interest were excised and subjected to electrospray‐ion trap tandem mass spectrometry (LCQ‐Deca, Finnigan).

### Plasmid Construction, Recombinant Protein Expression and Purification

His‐EBF2 plasmid was constructed by inserting the CDS of EBF2 into PET‐28a vector, transformed into *E. coli* BL21 (DE3), and cultured with isopropyl‐β‐D‐thiogalactoside (IPTG; Beyotime) at 16 °C for 12 h, until the optical density (OD600) reached 0.5–0.6. BL21 cells were collected and sonicated in cold PBS buffer, and His‐fusion proteins were purified with High Affinity Nickel beads (Genscript, Nanjing, China). The purity of His‐fusion proteins was evaluated by SDS‐PAGE.

### Microscale Thermophoresis (MST) Assay

Purified recombinant EBF2 proteins were labeled with Monolith NT‐647‐NHS. Labeled proteins were used at a concentration of 100 nm in PBS containing 0.05% Tween‐20 (pH 7.4). All the concentrations of H3, H3K4me1, and H3K4me3 peptides ranged from 10 nm to 500 µm. The combined solution of labeled proteins and peptides was incubated for 5 min and transferred into silicon‐treated capillaries. Thermophoresis was measured for 30 s on a NanoTemper Monolith NT.115 (NanoTemper Technologies GMBH, Germany) using LED power of 60% and 20%. Dissociation constants were calculated by NanoTemper Analysis 1.5.41 software using the mass action equation (Kd formula).

### Isothermal Titration Calorimetry (ITC) Assay

A MicroCal ITC‐200 system (Malvern Instruments Ltd., UK) was used for ITC assay. Briefly, the synthesized peptides (Genscript) and proteins were all subjected to extensive dialysis against PBS. Peptides at a concentration of 1 mm were loaded into the ITC syringe, and proteins at a concentration of 100 µm into the ITC cell. Then, every 2 µL of peptide was automatically injected into the cell at 25 °C. The results of binding isotherms were analyzed using the Origin 7.0 software package (Origin Lab).

### Immunofluorescence (IF) and Confocal Microscopy

IF was performed on 5 µm sections of tissue specimens following the manufacturer's protocol. PDAC and adjacent tissues were fixed with 4% formaldehyde for 24 h at room temperature. Having been washed three times with PBS containing 0.1% Triton X‐100, incubated with primary antibodies (H3K4me1 and EBF2) for 1 h at room temperature, washed again, incubated with secondary antibodies for 1 h at room temperature, stained with DAPI (Sigma, USA), and visualized by confocal scanning microscopy (Olympus FV10i, Japan).

### Protein Extraction and Western Blot Analysis

SW1990 and PANC‐1 cells were isolated using lysate buffer containing protease inhibitors. Samples containing equal amounts of protein were separated by SDS‐PAGE. After electrophoresis, the proteins were transferred to a PVDF membrane (Millipore, Billerica, MA, USA) and blocked with 5% skim milk for 2 h at room temperature. An overnight incubation of membranes was made at 4 °C with primary antibodies against KMT2D (1:1000; Affinity, China), EBF2 (1:1000; Affinity), and KLLN (1:1000; Abcam, USA). After washing with 1× TBST for three times, a 2 h incubation of membranes was implemented with horseradish peroxidase (HRP)‐gemeled Affinipure Goat Anti‐Rabbit IgG (H + L) (1:10000; ProteinTech, China) or Peroxidase‐gemeled Affinipure Goat Anti‐Mouse IgG (H+L) (1:10000; ProteinTech) at room temperature. Proteins were visualized by chemiluminescence using an ECL kit (Thermo Fisher).

### Co‐Immunoprecipitation Assay (Co‐IP)

Co‐immunoprecipitation assay was performed as described previously.^[^ ^61^ ^]^ Briefly, SW1990 cells were transfected with indicated constructs or treated with GSK‐LSD1, washed with cold phosphate buffered saline (PBS), and lysed with cold cell lysis buffer. Then, the lysates were incubated with appropriate specific antibodies or normal rabbit IgG at 4 °C overnight in constant rotation, followed by the addition of protein A/G Sepharose beads and incubation for 2 h at 4 °C. The beads were then washed five times by the lysis buffer. Bead‐bound proteins were resolved by SDS‐PAGE and detected by immunoblotting using indicated antibodies.

### RNA Isolation and Quantitative Real‐Time PCR (qPCR)

Total RNA was extracted from cultured cells using RNAiso plus (Takara, Japan). cDNA was synthesized from every 1 µg of total RNA in reverse transcription reactions with Hifair III 1st Strand cDNA Synthesis SuperMix for qPCR (Cat No. 11 141; Yeasen, Shanghai, China). qPCR was performed with Hieff qPCR SYBR Green Master Mix (Yeasen) using a Roche LightCycler 96 Real‐Time PCR System. Cycling conditions were set at 94 °C for 15 s, 60 °C for 1 min, and 72 °C for 30 s. Each reaction was performed in triplicate. The primer sequences were as follows: KMT2D‐F: 5′‐GCTGGCTGGTGAGGATAAAG‐3′; KMT2D‐R: 5′‐CAGTTACAGAGAGCACAACGC‐3′; EBF2‐F: 5′‐CATGTCATCAAGTCCCACCG‐3′; EBF2‐R: 5′‐TTACATCGGGGGTACAACAAG‐3′; KLLN‐F: 5′‐GTTGAGTGGAAAGTACGGAACG‐3’; KLLN‐R: 5′‐TGTGGGTGCTTGTGTAACCAG‐3′. β‐actin‐F: 5′‐CCTAGAAGCATTTGCGGTGG‐3′; β‐actin‐R: 5′‐GAGCTACGAGCTGCCTGACG‐3′.

### RNA Sequencing (RNA‐Seq) Assay

Total RNA was isolated from cells using RNAiso plus (Takara). The quantity and integrity of RNA were separately evaluated using the K5500 (Beijing Kaiao, China) and the Agilent 2200 TapeStation (Agilent Technologies, USA). Briefly, the mRNA was enriched by OligodT NEBNext Poly(A) mRNA Magnetic Isolation Module (NEB, USA), and then fragmented into ≈200 bp. Subsequently, the RNA fragments were subjected to first and second strand cDNA synthesis, followed by adaptor ligation and enrichment according to instructions of NEBNext Ultra RNA Library Prep Kit for Illumina. The purified library products were evaluated by the Agilent 2200 TapeStation and Qubit (Thermo Fisher Scientific, USA). The libraries were sequenced by Illumina (Illumina, USA) with paired‐end 150 bp (Ribobio, China). The clean reads were acquired after discarding low‐quality reads or those containing adapter and ploy‐N. HISAT2 was employed to calibrate clean reads to human reference genome hg38 with default parameters. HTSeq was used to convert aligned short reads into read counts for each gene model. Differential gene expression was assessed by DESeq2 using read counts as input.^[^ ^62^ ^]^ The Benjamini–Hochberg multiple test correction method was employed. Genes with significant upregulation under indicated conditions (fold change > 1.5, *p* < 0.05) were visualized using heat maps.^[^ ^63^ ^]^ RNA‐seq data sets were available (GEO accession number GSE237363).

### CCK‐8, EdU Incorporation, Colony Formation, and Transwell Assays

Cell proliferation was determined by Cell Counting Kit‐8 (CCK‐8, Yeasen) and EdU Cell Proliferation Assay kit (Ribobio, Guangzhou, China). Colony formation was observed by staining with 0.1% crystal violet (Sangon Biotechnologies Inc., Shanghai, China). Cell migration and invasion were assessed using 8 µm pore Transwell chambers with (for invasion assay) or without Matrigel (for migration assay). For migration assay, 5 × 10^4^ SW1990 or 3 × 10^4^ PANC‐1 cells were seeded into the upper chamber of the Transwell apparatus (Corning, USA) in serum‐free medium, and the medium supplemented with 10% FBS was added to the bottom chamber. For invasion assay, 10 × 10^4^ SW1990 or 6 × 10^4^ PANC‐1 cells were seeded into the upper Corning BioCoat Matrigel invasion chamber. After 24 h, the cells on the upper surface that had not passed through the polycarbonate filter were removed using a moistened cotton swab; the cells having migrated to the lower membrane surface were fixed in 100% methanol for 10 min, stained with 0.4% crystal violet for 15 min, and counted under a microscope (Nikon, Japan) at ×100 magnification. The cells were manually counted using Image J software. Three independent experiments were performed.

### Chromatin Immunoprecipitation (ChIP)

ChIP was performed with SW1990 cells using Cell Signaling Technology ChIP kit (CST, USA). Normal rabbit IgG served as the control. ChIP samples were analyzed by qPCR using the FastStart Universal SYBR Green Master. The primer sequences for ChIP were as follows: Primer 1‐F: 5′‐CTCCCGCCCGAGCCCACGG‐3′; Primer 1‐R: 5′‐AGGCGAGGGAGATGAGAGAC‐3′. Primer 2‐F: 5′‐CGTGTTGGAGGCAGTAGAAG‐3′; Primer 2‐R: 5′‐GGCACCTCCCGCTCCTGGAG‐3′. Primer 3‐F: 5′‐CTACTCAATATCCATTCTATG‐3′; Primer 3‐R: 5′‐CAACTTTGAACTGTATGTAG‐3′.

### ChIP‐Seq Data Processing and Analysis

ChIP‐seq was performed using modified ChIP and ChIP‐seq protocols previously described.^[^ ^64^ ^]^ ChIP samples were sequenced and analyzed for H3K4me1 and EBF2 by Shanghai Jiayin Biotechnology Co., Ltd (Shanghai, China). The Raw data of fastq format were first processed through in‐house perl scripts. Next, the clean data were obtained by removing reads containing adapter, reads containing ploy‐N, and low‐quality data from raw data. All the downstream analyses were based on high‐quality clean data. Then, using the bwa program, the clean reads were aligned to the reference genome sequences.^[^ ^65^ ^]^ ChIP‐seq data sets were available (GEO accession number GSE237364).

### Cleavage Under Targets and Tagmentation (CUT&Tag)

CUT&Tag was performed according to the instruction of Hyperactive Universal CUT & Tag Assay Kit for Illumina Pro (Cat No. TD904; Vazyme, Nanjing, China). TruePrep Index Kit V4 (Cat No. TD204; Vazyme) was used for index‐labeled DNA library preparation. Paired‐end sequencing was provided by LC Bio (Hangzhou, China).^[^ ^66^ ^]^ CUT&Tag data sets were available (GEO accession number GSE237365).

### Clinical Samples and Immunohistochemical (IHC) Staining

PDAC and tumor adjacent tissues were obtained from the Second Affiliated Hospital of Nanjing Medical University. Ninety pairs of ductal adenocarcinoma and adjacent normal pancreatic tissue sections (HPanA180Su03) were obtained from Shanghai Outdo Biotech (National Human Genetic Resources Sharing Service Platform with code No. XT20‐021, Shanghai, China), under the approval by the Ethics Committee of Shanghai Outdo Biotech. IHC staining was performed on paraffin‐embedded sections of biopsies from ductal adenocarcinoma patients and controls according to standard protocols (CST). Briefly, the sections were incubated with primary antibodies, including anti‐EBF2 (1:100 dilution; Affinity), anti‐KMT2D (1:100 dilution; Abcam), anti‐KLLN (1:100 dilution; Abcam), followed by incubation with horseradish peroxidase‐conjugated goat anti‐rabbit secondary antibodies. Antibody binding was visualized using a 2‐Solution DAB Kit (Invitrogen). All pancreatic cancer tissue sections were examined by two experienced pathologists, and the EBF2, KMT2D or KLLN stained in the tissue was scored independently using the H‐score system by two pathologists blinded to the clinical data. Rare discordance in scoring was resolved by re‐examination and consultation between the pathologists. The intensity of immunostaining (category A) was documented as 0–3: 0, negative; 1, weak; 2, moderate; 3, strong. For the Pearson correlation scatter plot of molecules in ductal adenocarcinoma, the H score was calculated by adding the multiplication product of the different staining intensities in category A (0–3) with the percentage of positive cells, i.e., H score (0–300 scale)  =  3 × (% at 3+) + 2 × (% at 2+) + 1 × (% at 1+). The clinical features of patients are listed in Additional file 1: Table S2 (Supporting Information). In the survival analysis, the overall survival was stratified by expression of the gene of interest and presented as Kaplan–Meier plots. The between‐group difference in the overall survival was evaluated by the log‐rank test. The correlation between EBF2, KMT2D, and KLLN expression in ductal adenocarcinoma was assessed via Pearson correlation analysis.

### Subcutaneous Tumorigenesis Model

BALB/c nude mice (4 weeks old) were purchased from the Animal Core Facility of Nanjing Medical University. Every 1 × 10^6^ SW1990‐luc cells (SW1990 cells labeled with luciferase) were suspended in 100 µL of PBS and Matrigel (Corning) mixture, then subcutaneously injected into the right forelimb of nude mice. The length and width of tumor were measured every 2 or 3 days. Tumor volume was calculated using the formula: Volume  =  0.5 × length × width^2^. Luciferase signals in nude mice were measured at the first and fourth week after injection. The mice were sacrificed at 30 days for in vivo proliferation assay. Mice were anesthetized with isoflurane gas and sacrificed by cervical dislocation. Tumor tissues of these mice were collected for further analyses.

### Lung Metastasis Model

BALB/c nude mice (5 weeks old) were purchased from the Animal Core Facility of Nanjing Medical University. About 1 × 10^5^ SW1990‐luc cells were suspended in 100 µL of PBS and injected into the caudal vein of nude mice. Luciferase signals in nude mice were measured weekly. The mice were anesthetized with isoflurane gas and sacrificed at 30 days by cervical dislocation for in vivo metastasis assay. Lung and liver samples were collected for further analyses.

### Patient‐Derived Xenograft (PDX) Model

A PDX model was established by implanting tumor fragments into immunodeficient nude mice. Tumor fragments were derived from a female PDAC patient with stage II ductal adenocarcinoma, who underwent surgery at the Second Affiliated Hospital of Nanjing Medical University. Informed consent was obtained from each patient, and all procedures involving human samples were approved by the Ethics Committee of the Second Affiliated Hospital of Nanjing Medical University.

### Bioluminescence Imaging

Luciferase signals from D‐luciferin (Cat No. MB1834; MeilunBio, Dalian, China) were measured using IVIS Spectrum (PerkinElmer, USA) at indicated concentrations. The luciferase signal activity was quantitated using the software provided by the manufacturer.

### Statistical Analysis

Statistical analysis was implemented on the GraphPad Prism 8.0 software (GraphPad Inc., La Jolla, CA, USA). Unless otherwise specified, data were collected from at least three independent experiments and expressed as mean ±SEM. Two‐group comparison of experiments data was accomplished through the Student's *t*‐test, and multiple‐group comparison by the ANOVA test. One‐way ANOVA was used to compare data of more than two groups under one treatment, whereas two‐way ANOVA to compare data of more than two groups under more than one treatment. Survival curves were constructed using the Kaplan–Meier method and analyzed by the log‐rank test. The correlations analysis was based on Pearson's correlation. The sample size of each experimental group was shown in each figure as the number of dots. *p* < 0.05 was considered statistically significant: ^*^ *p* < 0.05, ^**^ *p* < 0.01.

### Ethics Approval and Consent to Participate

Written informed consent was obtained from all patients. The study was approved by the Ethics Committee of Nanjing Medical University (2021‐KY‐096‐01 and SHYJS‐CP‐1901009).

## Conflict of Interest

The authors declare no conflict of interest.

## Supporting information

Supporting Information

## Data Availability

The datasets used in the current study are available from the corresponding author on reasonable request.
