# Supplementary material for: EBF2 Links KMT2D‐Mediated H3K4me1 to Suppress Pancreatic Cancer Progression via Upregulating KLLN
Source: Adv Sci (Weinh). 2023 Nov 28;11(2):2302037. doi: 10.1002/advs.202302037 (PMC10787067; doi:10.1002/advs.202302037)
Supplement: Supplementary file 1 — Supporting Information [file ADVS-11-2302037-s001.pdf]

ADVANCED SCIENCE
Open Access

Supporting Information

for *Adv. Sci.*, DOI 10.1002/advs.202302037

EBF2 Links KMT2D-Mediated H3K4me1 to Suppress Pancreatic Cancer Progression via Upregulating KLLN

Bing Yao, Mengying Xing, Shixin Meng, Shang Li, Jingwan Zhou, Ming Zhang, Chen Yang, Shuang Qu, Yucui Jin, Hongyan Yuan, Ke Zen* and Changyan Ma**

Supporting Information for

EBF2 links KMT2D-mediated H3K4me1 to suppress pancreatic cancer progression via upregulating KLLN

Bing Yao [#*1], Mengying Xing[#1], Shixin Meng[1], Shang Li[1], Jingwan Zhou[1], Ming Zhang[1], Chen Yang[2], Shuang Qu[3], Yucui Jin[1], Hongyan Yuan[4], Ke Zen[*2], Changyan Ma[*1, 5]

File list:

1. Table S1
2. Table S2
3. Figure S1
4. Figure S2
5. Figure S3
6. Figure S4
7. Figure S5
8. Figure S6

1. Table S1. List of proteins identified by Mass Spectrometry

No.	Protein names	Score	Intensity H3	Intensity H3K4me1	No.	Protein names	Score	Intensity H3	Intensity H3K4me1
1	HTATSF1	323.31	0	7896300000	267	SRPRB	6.2092	0	6369800
2	ACIN1	323.31	0	4538700000	268	KNOP1	6.0187	0	11633000
3	AP3B1	323.31	0	7022400000	269	VARS	5.9202	0	23386000
4	SRRM2	311.46	0	4108200000	270	USP15	5.8594	0	189950000
5	MARS	294.58	0	1815900000	271	NPM3	5.7768	0	130200000
6	YBX1	289.03	0	982660000	272	AKAP8L	5.6751	0	12950000
7	RARS	285.78	0	3337800000	273	RBM28	5.6321	0	93962000
8	UBR4	277.05	0	2466900000	274	SRCAP	5.4613	0	84452000
9	EIF3C	249.44	0	8652500000	275	PGAM5	5.2471	0	177960000
10	HNRNPUL1	246.43	0	9168800000	276	CEBPZ	5.1627	0	96981000
11	EIF3L	237.02	0	1299900000	277	MRPS34	4.9979	0	171250000
12	SSBP1	232.46	0	1380200000	278	SKIV2L2	4.9587	0	10405000
13	EIF3A	231.93	0	3973000000	279	SEC63	4.9458	0	68709000
14	EBF2	228.34	0	1117500000	280	MRS2	4.944	0	43330000
15	SQSTM1	201.95	0	93569000	281	KDM5B	4.9246	0	54916000
16	DDX21	191.54	0	1351800000	282	MRPL46	4.9129	0	38520000
17	MRPL44	187.09	0	482350000	283	PCNA	4.8947	0	132910000
18	ZC3H18	177.01	0	2525800000	284	SRPK2	4.8922	0	215950000
19	PNN	176.74	0	6795000000	285	RECQL	4.8399	0	158410000
20	CTR9	176.34	0	3612700000	286	NUP160	4.6445	0	32284000
21	UBR5	173.91	0	1156600000	287	HP1BP3	4.6364	0	116600000
22	ANP32B	170.49	0	7289400000	288	GPATCH8	4.6311	0	97992000
23	UBTF	167.27	0	3916500000	289	EIF6	4.6221	0	9906100
24	SUPT16H	165.02	0	6535100000	290	XPC	4.599	0	15627000
25	ZC3HAV1	162.87	0	666660000	291	BCLAF1	4.4624	0	264370000
26	CSNK2A1	161.86	0	2541600000	292	RNPS1	4.4449	0	109410000
27	RPA1	159.91	0	1151900000	293	TGFBR3	4.4394	0	60024000
28	SART3	156.04	0	9491300000	294	RPL18	4.427	0	1134900000
29	PELP1	144.49	0	1705500000	295	CHD8	4.4223	0	220130000
30	VPRBP	142.38	0	2635900000	296	MRPL40	4.3467	0	9106800
31	CHD1	140.57	0	4252600000	297	ETF1	4.3146	0	161510000
32	SUPT5H	139.75	0	575100000	298	FXR2	4.3006	0	10034000
33	YTHDC2	137.06	0	1013900000	299	VDAC3	4.2833	0	148810000
34	TCOF1	136.86	0	1.3374E+10	300	POLR2H	4.2578	0	30638000
35	ANP32E	135.75	0	601580000	301	GADD45G	4.2353	0	154270000
36	SRP19	134.87	0	1279700000	302	CCNT1	4.2337	0	8169600
37	POP1	134.51	0	1630400000	303	EIF3H	4.1855	0	65074000
38	MRPL21	126.13	0	952400000	304	SFXN1	4.15	0	11507000
39	ADNP	121.54	0	560900000	305	PSMC5	4.1154	0	27908000
40	NOLC1	120.13	0	5982000000	306	FBL	4.0944	0	135770000
41	MRPS9	118.37	0	1088900000	307	SDC4	4.0302	0	399050000
42	OSBPL3	118.29	0	600260000	308	RANBP2	4.0103	0	47693000
43	MRPS35	114.54	0	318450000	309	GNL3	3.9746	0	38386000
44	SRP72	112.5	0	777370000	310	PRPF4B	3.9183	0	34627000
45	MRPL49	111.58	0	387310000	311	POLR2A	3.8829	0	40259000
46	UPF1	104.58	0	792370000	312	SAP18	3.8462	0	1718400000
47	MYBBP1A	104.45	0	444120000	313	FAM120A	3.8346	0	11234000

48	HNRNPA3	103.28	0	1934600000	314	MRPS22	3.8162	0	92141000
49	MOV10	102.95	0	533520000	315	RPL27A	3.8007	0	667330000
50	ZFR	101.51	0	240470000	316	ABCF2	3.7097	0	84742000
51	ILF2	100.63	0	1817800000	317	DDX24	3.6927	0	96521000
52	YTHDC1	100.62	0	1189900000	318	RALY	3.6885	0	165630000
53	KANK1	100.29	0	204870000	319	NOP56	3.6663	0	48512000
54	SSRP1	100.06	0	3678500000	320	SNRNP70	3.6617	0	48567000
55	RPL7A	97.91	0	4769100000	321	RRP1	3.6333	0	47194000
56	MRPS7	97.44	0	781560000	322	NARS2	3.5733	0	202130000
57	SRP14	96.11	0	806630000	323	RCC2	3.5239	0	107540000
58	RPL3;rpl3	95.599	0	794780000	324	HSDL2	3.4933	0	11551000
59	RPL6	94.284	0	8141500000	325	PURB	3.4897	0	29084000
60	SDC1	92.562	0	339690000	326	MKI67	3.4549	0	6825700
61	MRPS31	91.975	0	400000000	327	EIF3G	3.447	0	7627500
62	GEMIN5	89.707	0	449550000	328	TRIP12	3.429	0	27239000
63	BMS1	88.833	0	882430000	329	SNRPE	3.411	0	190350000
64	CHD4	87.379	0	1057900000	330	CMTR1	3.3932	0	67189000
65	CSNK2B	81.872	0	1079400000	331	SRSF1	3.3601	0	79616000
66	MRPL9	80.942	0	987370000	332	GARS	3.3145	0	87683000
67	LYAR	78.947	0	317120000	333	CYFIP1	3.2795	0	10915000
68	MRPL32	78.456	0	113670000	334	GNL1	3.2446	0	37484000
69	DAP3	76.701	0	1093900000	335	KAT7	3.2375	0	41303000
70	LARP1	75.466	0	2459900000	336	CYR61	3.1639	0	77535000
71	ALYREF	75.302	0	123690000	337	MRPL27	3.1597	0	405530000
72	CSNK2A2	74.896	0	686270000	338	CWC22	3.1341	0	219580000
73	TOP1	73.12	0	1988000000	339	NOC3L	3.113	0	16120000
74	EIF3D	73.023	0	136400000	340	RUVBL2	3.1087	0	23569000
75	IPO9	72.8	0	285170000	341	TARS	3.108	0	34473000
76	YBX3	71.661	0	91788000	342	DDX27	3.0674	0	47628000
77	XRCC6	70.34	0	1788800000	343	FGF2	3.0607	0	95695000
78	MTDH	69.724	0	107060000	344	MRPL30	3.0043	0	82321000
79	SRSF4	69.634	0	1304800000	345	ARID4B	2.9889	0	19855000
80	MEPCE	68.283	0	1310500000	346	SRSF6	2.9869	0	231750000
81	SNRPA1	67.446	0	1831000000	347	SMC4	2.9812	0	12317000
82	RPL17	67.111	0	707290000	348	MRPL24	2.9591	0	375890000
83	PAF1	66.293	0	1257300000	349	MRPS5	2.8542	0	45555000
84	MRPL37	65.128	0	1266000000	350	STT3A	2.8342	0	18287000
85	AIMP2	60.102	0	1394100000	351	AGO2	2.8341	0	42849000
86	HUWE1	59.885	0	241240000	352	HARS2	2.7924	0	24467000
87	SIN3A	59.318	0	147780000	353	XRN1	2.7891	0	21950000
88	DDB1	58.246	0	391740000	354	MCM3	2.7687	0	55361000
89	RPL9	57.67	0	292860000	355	PUF60	2.7179	0	27475000
90	HBS1L	57.319	0	119680000	356	SMARCAI	2.7146	0	42301000
91	SNRPD1	56.654	0	662720000	357	THOC2	2.6787	0	26731000
92	C1QBP	56.644	0	1897700000	358	DDB2	2.6122	0	76330000
93	CHAF1A	56.542	0	149660000	359	AFF4	2.5955	0	65078000
94	MRPS10	54.7	0	151040000	360	HNRNPDI	2.5913	0	155110000
95	MRPL23	54.582	0	306380000	361	CENPV	2.5591	0	47536000
96	KPNA1	53.375	0	74055000	362	EIF2B5	2.5085	0	124100000
97	DARS	52.994	0	3628000000	363	MTA2	2.4967	0	42633000
98	NKTR	52.312	0	240410000	364	HSD17B10	2.4804	0	21611000

99	ATRX	51.138	0	427210000	365	POP7	2.4757	0	74688000
100	U2AF1	50.675	0	124020000	366	RPL36A	2.4734	0	354030000
101	LARP7	50.621	0	732800000	367	KPNA3	2.4682	0	53524000
102	MRPL13	50.194	0	625830000	368	TSR1	2.4667	0	46266000
103	EIF5B	49.981	0	1982500000	369	WDR77	2.4553	0	29751000
104	UBE2O	48.515	0	669950000	370	MRPS2	2.446	0	54458000
105	HNRNPUL2	47.353	0	81489000	371	OSTC	2.4153	0	27290000
106	MRPS30	47.289	0	335170000	372	ANKRD17	2.4108	0	72376000
107	RTF1	46.344	0	631090000	373	IPO5	2.3681	0	15613000
108	LUC7L3	45.969	0	30206000	374	SON	2.3402	0	44968000
109	HNRNPH3	45.779	0	159490000	375	DIEXF	2.339	0	35418000
110	SLC25A11	44.991	0	38404000	376	DYNLL1	2.3234	0	53565000
111	RRP1B	44.756	0	30244000	377	MRPS24	2.3206	0	108190000
112	TRIM26	44.574	0	1025200000	378	EIF2AK2	2.3187	0	25113000
113	CHD9	43.543	0	229820000	379	MYO1B	2.315	0	30724000
114	MRPL38	42.445	0	1050300000	380	POLR2B	2.3092	0	20610000
115	ILF3	41.931	0	1355400000	381	PRPF8	2.2781	0	30614000
116	MRPL1	41.059	0	247210000	382	ANKRD28	2.2702	0	9595400
117	MRPL4	39.04	0	520120000	383	SART1	2.2564	0	29376000
118	CHD2	38.685	0	58144000	384	KDM5A	2.2454	0	37390000
119	NAT10	38.676	0	284330000	385	SUPT6H	2.2369	0	19793000
120	MYO10	38.367	0	29476000	386	MRPL17	2.2165	0	205110000
121	NRD1	37.853	0	650390000	387	CDC27	2.1971	0	53137000
122	EHMT2	36.75	0	524760000	388	MRPL48	2.19	0	27915000
123	RPP30	36.684	0	101650000	389	SNRPB	2.1489	0	442710000
124	DDX20	36.006	0	107100000	390	CD44	2.1317	0	61177000
125	CHCHD2	35.844	0	414570000	391	DIS3L2	2.0858	0	14929000
126	U2AF2	35.188	0	223580000	392	IGF2BP3	2.0529	0	16730000
127	THUMPD1	35.08	0	89588000	393	FARSA	2.0453	0	21695000
128	AIMP1	34.59	0	1619300000	394	RBMS2	2.045	0	28918000
129	RBMX	34.409	0	974150000	395	DRG1	2.0273	0	21009000
130	GTF3C5	33.651	0	17588000	396	RPA2	2.0053	0	37721000
131	MRPL39	33.504	0	718740000	397	BPTF	1.9867	0	38418000
132	RPL32	33.467	0	22700000	398	CSE1L	1.9861	0	19616000
133	MRPS27	33.15	0	284230000	399	ELAVL1	1.9392	0	63875000
134	PSMD3	32.721	0	252570000	400	HIST1H1D	1.9357	0	50220000
135	HIST1H1C	32.678	0	7668100000	401	LUC7L2	1.9329	0	42209000
136	MCM4	32.474	0	128500000	402	ZRANB2	1.8425	0	24065000
137	PPP6R3	32.027	0	85889000	403	EFTUD2	1.84	0	42951000
138	CHORDC1	31.483	0	53583000	404	COPB1	1.797	0	5685900
139	PCM1	31.006	0	356880000	405	SURF4	1.7851	0	43379000
140	NCBP1	29.141	0	761070000	406	SRRT	1.7831	0	35492000
141	HLTF	29.004	0	158150000	407	ZNF622	1.7801	0	33485000
142	TPR	28.744	0	79484000	408	XPOT	1.7611	0	20769000
143	PELO	28.597	0	69560000	409	AHCYL2	1.7609	0	10464000
144	RAB5C	27.421	0	76752000	410	STK26	1.7518	0	7960300
145	UHRF1	27.349	0	193760000	411	RGPD3	1.7044	0	19086000
146	LARS	26.795	0	447230000	412	HNRNPL	1.7008	0	42192000
147	RSL1D1	26.481	0	308140000	413	KHDRBS1	1.6877	0	97730000
148	CDK9	26.264	0	42480000	414	RBBP6	1.6833	0	9479400
149	LSM2	26.011	0	57902000	415	VKORC1	1.6714	0	14044000

150	BRIX1	24.804	0	268860000	416	ACTR2	1.6711	0	18076000
151	XRN2	24.146	0	325110000	417	WDR3	1.6503	0	23264000
152	CDK11B	23.995	0	2544800000	418	U2SURP	1.6422	0	28839000
153	EBNA1BP2	23.307	0	406210000	419	MRPL37	1.6013	0	11499000
154	AHSA1	23.025	0	42713000	420	ATP5J2	1.5952	0	12636000
155	EIF3I	22.974	0	830570000	421	EWSR1	1.5843	0	15510000
156	RPL23A	22.934	0	4062200000	422	SBDS	1.5319	0	15834000
157	RPL28	21.871	0	692380000	423	DCAF8	1.5234	0	16023000
158	DHX36	21.532	0	38176000	424	PWP1	1.4951	0	20134000
159	MRPL45	21.144	0	325410000	425	RPL29	1.481	0	158670000
160	THRAP3	21.033	0	131920000	426	MRPL50	1.4798	0	31933000
161	MRPL43	20.593	0	375610000	427	HDAC1	1.4541	0	147130000
162	MRPL2	20.395	0	462460000	428	LSM6	1.4444	0	39537000
163	DKC1	20.321	0	370350000	429	PKN2	1.4279	0	7552600
164	MYO1C	20.132	0	124860000	430	RPS15	1.4235	0	247630000
165	H2AFY	20.078	0	53080000	431	EMG1	1.4163	0	45913000
166	RPL35A	19.183	0	1649700000	432	ZUBR1	1.4071	0	51455000
167	HDGFRP2	19.048	0	124480000	433	PYGB	1.3982	0	30335000
168	PTCD3	19.041	0	778300000	434	VAPA	1.393	0	53288000
169	EIF2A	18.664	0	333350000	435	SLC16A3	1.3902	0	19643000
170	LEO1	18.282	0	881610000	436	MRPL47	1.385	0	177910000
171	MYO1E	18.018	0	205930000	437	PHRF1	1.3832	0	62224000
172	TCOF1	17.804	0	68357000	438	JMJD6	1.3644	0	8421700
173	EMD	17.688	0	110550000	439	SF3B2	1.3634	0	25601000
174	TRA2B	17.333	0	461930000	440	RTCB	1.3582	0	39156000
175	MRTO4	17.317	0	162270000	441	TRA2A	1.3254	0	57130000
176	TSPYL2	17.132	0	193250000	442	MGST1	1.3221	0	12387000
177	TRMT6	17.132	0	41169000	443	RAB6A	1.3148	0	10271000
178	G3BP1	17.054	0	282100000	444	TBC1D31	1.2893	0	168040000
179	ATAD2	16.99	0	161340000	445	PDS5B	1.2628	0	13142000
180	PPP2R2A	16.902	0	67883000	446	TGS1	1.2489	0	11437000
181	GATAD2B	16.87	0	75360000	447	PDCD11	1.2489	0	6719800
182	PDCD6IP	16.367	0	84939000	448	CDC5L	1.2378	0	49167000
183	SDC2	16.269	0	704170000	449	SLC16A1	1.2159	0	35473000
184	KIAA1429	16.13	0	82081000	450	MRPL52	1.2064	0	49872000
185	WDR5	16.093	0	905550000	451	RAB14	1.196	0	18492000
186	USP39	15.99	0	98228000	452	APEX1	1.1939	0	13543000
187	SRSF10	15.93	0	91394000	453	RB1CC1	1.1806	0	85266000
188	RPS26	15.527	0	1552200000	454	RAC1	1.178	0	29761000
189	SETD1B	15.384	0	50299000	455	ANAPC7	1.1767	0	13942000
190	MRPL19	14.915	0	1268800000	456	SNRPA	1.1711	0	115620000
191	SETD1A	14.258	0	65851000	457	ANP32A	1.1646	0	449110000
192	C7orf50	14.241	0	28687000	458	SAP30BP	1.1623	0	16914000
193	RFC1	14.132	0	72707000	459	RPP40	1.1601	0	18098000
194	EIF3E	13.873	0	566310000	460	EPB41L1	1.1431	0	10636000
195	SMARCA5	13.66	0	339880000	461	CDK2	1.1372	0	24090000
196	NHP2L1	13.494	0	44145000	462	STRAP	1.1281	0	38476000
197	MRPL15	13.437	0	793950000	463	SLC25A13	1.1231	0	8883900
198	TRIM44	12.751	0	20472000	464	DDX47	1.102	0	25140000
199	NKRF	12.731	0	372750000	465	DDX50	1.0847	0	13237000
200	CFAP20	12.709	0	247120000	466	TRIM25	1.0296	0	26349000

201	GEMIN4	12.592	0	206330000	467	RBBP5	1.029	0	56774000
202	NOP2	12.503	0	182870000	468	SUDS3	1.0202	0	18576000
203	IWS1	12.449	0	1924500000	469	APLP2	1.0095	0	20417000
204	AP3D1	12.246	0	285840000	470	FLII	1.0044	0	54608000
205	MRPS18B	12.141	0	106150000	471	ZZEF1	0.99944	0	7008900
206	COPA	11.884	0	91198000	472	YARS	0.99138	0	10924000
207	MORF4L2	11.817	0	87947000	473	RPS10	0.98946	0	16148000
208	RRP9	11.775	0	249040000	474	DROSHA	0.9851	0	23753000
209	MSH6	11.611	0	90444000	475	PHF8	0.96102	0	8176600
210	EIF3G	11.514	0	440120000	476	BRD2	0.94978	0	14359000
211	RBM39	11.339	0	50703000	477	FTSJ3	0.93238	0	133180000
212	BUB3	11.134	0	105580000	478	ABCF3	0.92995	0	5264900
213	RPL27	10.83	0	403110000	479	DDX19A	0.91998	0	22568000
214	KPNA4	10.714	0	375760000	480	ATP5L	0.91901	0	22361000
215	RPS24	10.695	0	181830000	481	OLA1	0.91591	0	77902000
216	HIST1H1B	10.668	0	1130900000	482	TLN2	0.91357	0	157200000
217	TROVE2	10.663	0	193260000	483	USP7	0.91171	0	23650000
218	HIST1H1A	10.064	0	69507000	484	MRPS17	0.91035	0	20804000
219	USP34	10.027	0	134710000	485	H1F0	0.90818	0	176040000
220	PRMT5	9.8984	0	82474000	486	CCPG1	0.90635	0	27975000
221	PABPN1	9.8053	0	45860000	487	CHERP	0.89254	0	18251000
222	NOC2L	9.7955	0	955940000	488	RPP25L	0.89134	0	10917000
223	MRPL3	9.6545	0	388970000	489	NAA10	0.89128	0	12172000
224	EEF1E1	9.5625	0	500340000	490	PSMD5	0.89026	0	6231200
225	PPP1R12A	9.4533	0	232110000	491	SNRPF	0.8777	0	50340000
226	NIFK	9.2011	0	194060000	492	CAPN13	0.87264	0	38295000
227	SRPK1	9.0009	0	165410000	493	LARP4	0.86611	0	6340000
228	ASPH	8.9914	0	42969000	494	METAP2	0.85846	0	44724000
229	NCAPH	8.9325	0	72607000	495	ANK3	0.85588	0	255060000
230	PRPF19	8.7914	0	259950000	496	MRPL34	0.8529	0	43214000
231	PPM1G	8.6643	0	238980000	497	ICT1	0.84966	0	260530000
232	DNTTIP2	8.6196	0	51143000	498	MRPL33	0.84339	0	26026000
233	DHX57	8.5614	0	65578000	499	CAV1	0.84157	0	53113000
234	BCR	8.5399	0	52712000	500	SDE2	0.83853	0	714480000
235	MRPL28	8.4658	0	436970000	501	ATAD3A	0.83604	0	6033900
236	EHMT1	8.313	0	363160000	502	MTCH2	0.82464	0	4415300
237	RAB21	8.2978	0	44536000	503	MLLT1	0.82284	0	21763000
238	ZCCHC10	8.2069	0	74648000	504	HERC2	0.82023	0	8769200
239	GPC1	8.1677	0	281460000	505	CIRBP	0.80148	0	20086000
240	PRPS1	8.1305	0	86873000	506	RBM3	0.79563	0	21237000
241	TRMT61A	8.1067	0	247350000	507	DDX23	0.76751	0	9385600
242	LBR	7.9991	0	34970000	508	ESYT1	0.766	0	8163200
243	WIZ	7.9716	0	60601000	509	MRPS18A	0.76232	0	187570000
244	SNRPD2	7.964	0	1367000000	510	GTPBP4	0.76128	0	31063000
245	CENPB	7.9572	0	302150000	511	MORC3	0.75093	0	10826000
246	MRPL20	7.8099	0	673180000	512	BIRC2	0.746	0	10420000
247	NCBP2	7.8038	0	178110000	513	SDAD1	0.73938	0	10724000
248	MRPS25	7.7768	0	55992000	514	KANK2	0.72957	0	23980000
249	POP4	7.7508	0	23946000	515	MAP2K3	0.72537	0	12829000
250	RANGAP1	7.6909	0	32277000	516	RPP25	0.7242	0	57526000
251	CTDP1	7.6488	0	9107100	517	CDK18	0.70898	0	283270000

252	CHD3	7.5987	0	167930000	518	MAP2K2	0.70599	0	7107200
253	PICALM	7.5079	0	5716300	519	CSNK1A1	0.69604	0	100810000
254	TMEM33	7.3898	0	48582000	520	MRPS23	0.69348	0	22728000
255	CMAS	7.3057	0	72763000	521	RPL36	0.68738	0	61609000
256	ESF1	7.1547	0	105780000	522	ADAR	0.68158	0	6643300
257	NOP58	7.1129	0	93309000	523	DST	0.68073	0	11345000
258	DDX18	7.0385	0	67938000	524	ARL2	0.66001	0	7923500
259	EIF3K	7.037	0	66541000	525	SSR3	0.65757	0	28405000
260	IGF2BP1	6.8127	0	71858000	526	CDC23	0.65467	0	21910000
261	DHFR	6.6955	0	45311000	527	DYNC1H1	0.65256	0	5615700
262	SRSF7	6.6158	0	349980000	528	GATAD2A	0.6523	0	9298600
263	H1FX	6.5894	0	461120000	529	CARD18	0.64474	0	14770000
264	MAP4	6.5123	0	152000000	530	ARHGEF1(	0.63716	0	12303000
265	MRPL22	6.4563	0	395150000	531	MRPL35	0.62419	0	18545000
266	PURA	6.2532	0	37329000					

2. Table S2. Clinicopathologic characteristics of EBF2 and KMT2D expression in PCa patients

Characteristics	Cases	IHC score of EBF2		IHC score of KMT2D	
		Mean±s.d.	*P* value[a]	Mean±s.d.	*P* value[a]
Age					
> 50	73	90.89 ± 6.556	0.4413	81.16 ± 5.558	0.6513
≤50	17	102.9 ± 15.88		87.06 ± 12.51	
Grade					
I-II	53	111.7 ± 7.903	0.0002	100.3 ± 8.1	0.0135
III-IIII	37	66.62 ± 7.752		147.6 ± 12.2	
Tumor Size					
T1- T2	44	117.6 ± 9.506	<0.0001	105.6 ± 7.561	<0.0001
T3-T4	45	69.56 ± 6.111		59.56 ± 5.032	
Lymph node status					
N0	32	116.1 ± 12.03	0.0017	100.2 ± 9.2.06	0.0018
N1-2	56	77.5 ± 5.800		69.11 ± 5.073	
Metastasis					
M0	66	103.3 ± 7.437	0.0050	88.48 ± 6.344	0.0414
M1	24	65.21 ± 7.805		65.21 ± 6.486	
Survival					
Yes	7	224.3 ± 18.50	<0.0001	180.7 ± 14.24	<0.0001
No	83	82.11 ± 4.720		73.98 ± 4.257	

[a] *P* values were derived using Student's *t*-test to compare values for the two parameters in each category.

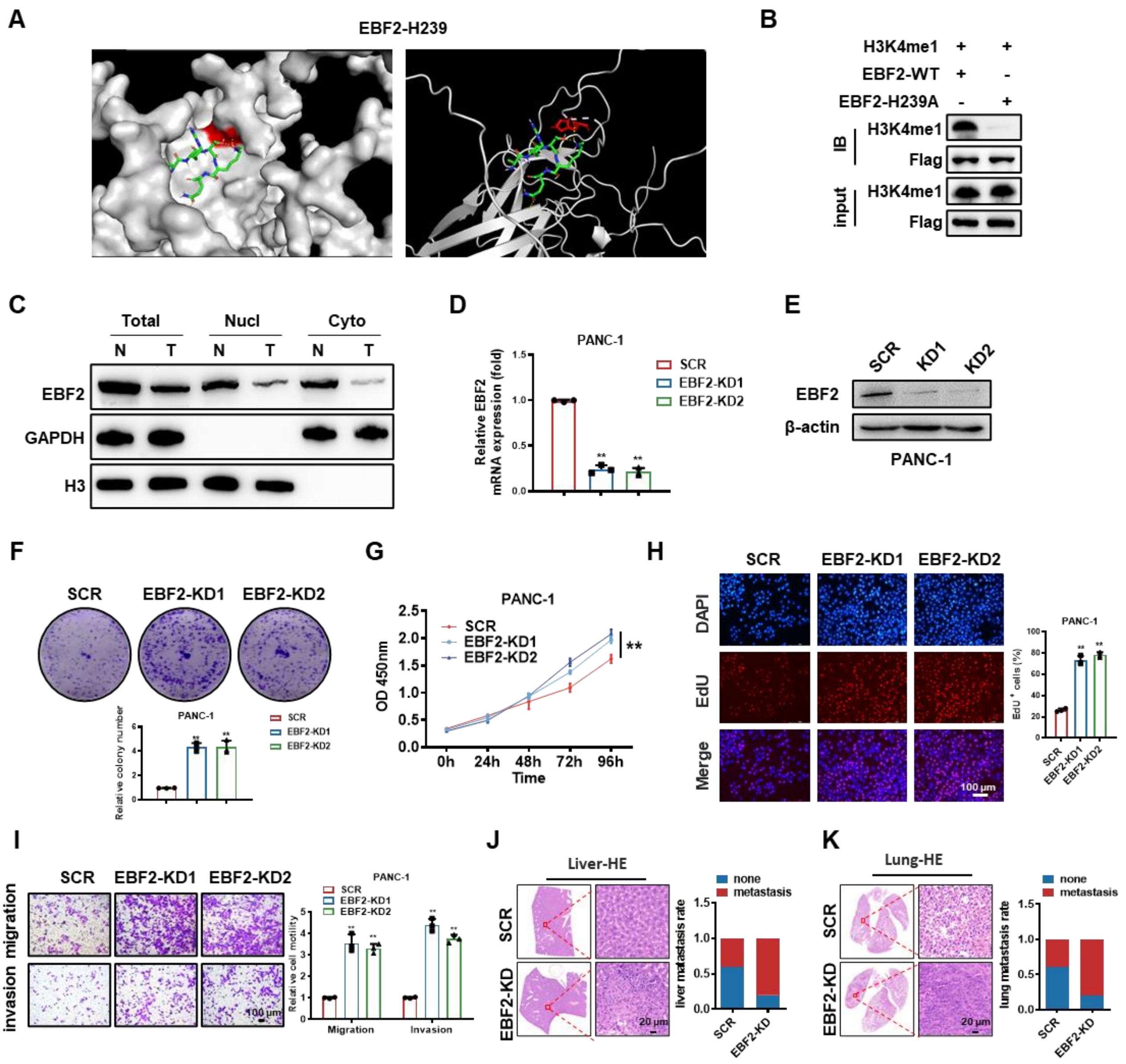

3. Figure S1. EBF2 inhibits PADC cell proliferation and metastasis *in vitro* and *in vivo*. A) Overall structure of the EBF2-H3K4me1 complex. Gray, EBF2; red, His239 (H239) of EBF2; green, a H3K4me1-containing histone H3 tail peptide. B) Co-IP assay to determine the interactions between Flag-tagged EBF2-WT or EBF2-H239A and H3K4me1 in SW1990 cells. C) Cellular distribution of EBF2 in normal pancreatic and PC tissue. D-E) The mRNA (D) and protein expression (E) of EBF2 in EBF2-depleted PANC-1 cells verified by qPCR and Western blot assays. Data in D are presented as mean ± SEM, ***P*<0.01 by one-way ANOVA test. F-I) Colony-formation (F), CCK-8 (G), EdU incorporation (H) and Transwell assays (I) assessed the effects of EBF2 knockdown on proliferation, migration and invasion of PANC-1 cells *in vitro*. Data in F-I are presented as mean ± SEM, ***P*<0.01 by two-way ANOVA test (G) and one-way ANOVA test (F, H and I). J-K) H&E staining showed that both liver (J) and lung metastasis rates (K) were increased in mice injected with EBF2-KD SW1990-luc cells. Scale bar, 20μm.

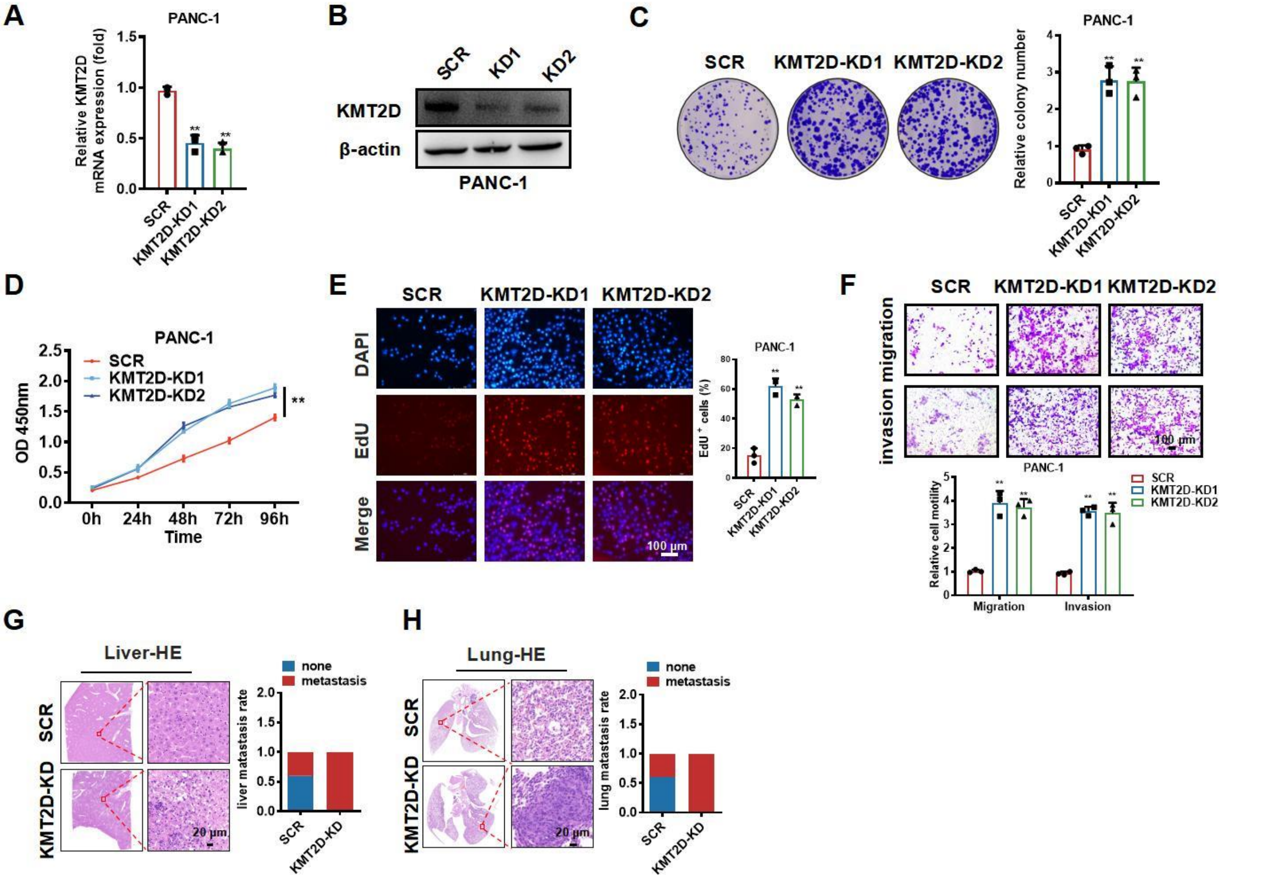

4. Figure S2. KMT2D inhibits cell proliferation and metastasis *in vitro* and *in vivo*. A-B) The mRNA (A) and protein (B) levels of KMT2D in KMT2D-depleted PANC-1 cells verified by qPCR and Western blot assays. Data in A are presented as mean ± SEM, ***P*<0.01 by one-way ANOVA test. C-F) Colony-formation (C), CCK-8 (D), EdU incorporation (E) and Transwell (F) assays assessed the effects of KMT2D knockdown on proliferation, migration and invasion of PANC-1 cells *in vitro*. Data in C-F are presented as mean ± SEM, ***P*<0.01 by two-way ANOVA test (D) and one-way ANOVA test (C, E and F). G-H) H&E staining showed that both liver (G) and lung (H) metastasis rates increased in mice injected with KMT2D-KD SW1990-luc cells. Scale bar, 20μm.

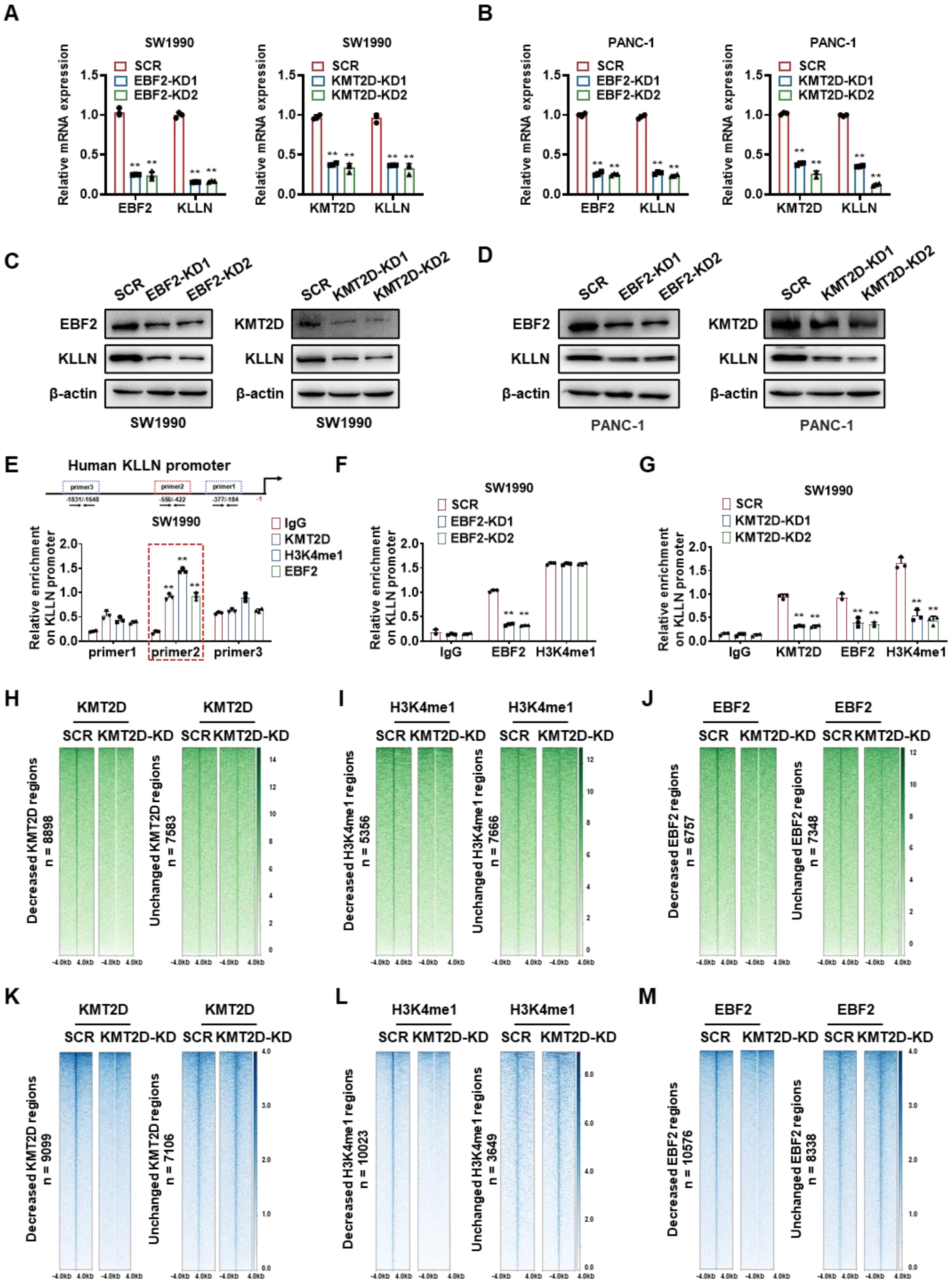

5. Figure S3. KLLN is a common transcriptional target of KMT2D and EBF2 in PDAC cells. A-B) qRT-PCR analysis of KLLN mRNA level in EBF2 or KMT2D-depleted SW1990 (A) and PANC-1 (B) cells. Data in A-B are presented as mean ± SEM, ***P*<0.01 by one-way ANOVA test. C-D) Western blot analysis of KLLN

protein level in EBF2 or KMT2D-depleted SW1990 (C) and PANC-1 (D) cells. E) ChIP analysis of KMT2D, H3K4me1 and EBF2 binding to the promoter region of KLLN gene in SW1990 cells. F) ChIP-qPCR analysis of EBF2 and H3K4me1 bindings to the TSS and promoter regions of KLLN in SCR, EBF2-KD1 and EBF2-KD2 SW1990 cells. G) ChIP-qPCR analysis of KMT2D, EBF2 and H3K4me1 bindings to the TSS and promoter regions of KLLN in SCR, KMT2D-KD1 and KMT2D-KD2 SW1990 cells. Data in E-G are presented as mean ± SEM, $^{**}P<0.01$ by Student's *t*-test (E) and one-way ANOVA test (F and G). H) Heatmap of input normalized KMT2D ChIP signal in control (SCR), KMT2D-KD over 8898 distal KMT2D regions with decreased signals in KMT2D-KD and 7583 distal KMT2D regions with invariable signals, with regions sorted by strength of KMT2D signal. I) Heatmap of input normalized H3K4me1 ChIP signal in SCR and KMT2D-KD over 5356 distal H3K4me1 regions with decreased signals in KMT2D-KD and 7666 distal H3K4me1 regions with invariable signals, with regions sorted by strength of H3K4me1 signal. J) Heatmap of input normalized EBF2 ChIP signal in SCR and KMT2D-KD over 6757 distal EBF2 regions with decreased signals in KMT2D-KD and 7348 distal EBF2 regions with invariable signals, with regions sorted by strength of EBF2 signal. K) Heatmap of KMT2D CUT&Tag signal in SCR and KMT2D-KD with regions sorted by strength of KMT2D signal. L) Heatmap of H3K4me1 CUT&Tag signals in SCR and KMT2D-KD with regions sorted by strength of H3K4me1 signal. M) Heatmap of EBF2 CUT&Tag signals in SCR and KMT2D-KD with regions sorted by strength of EBF2 signal.

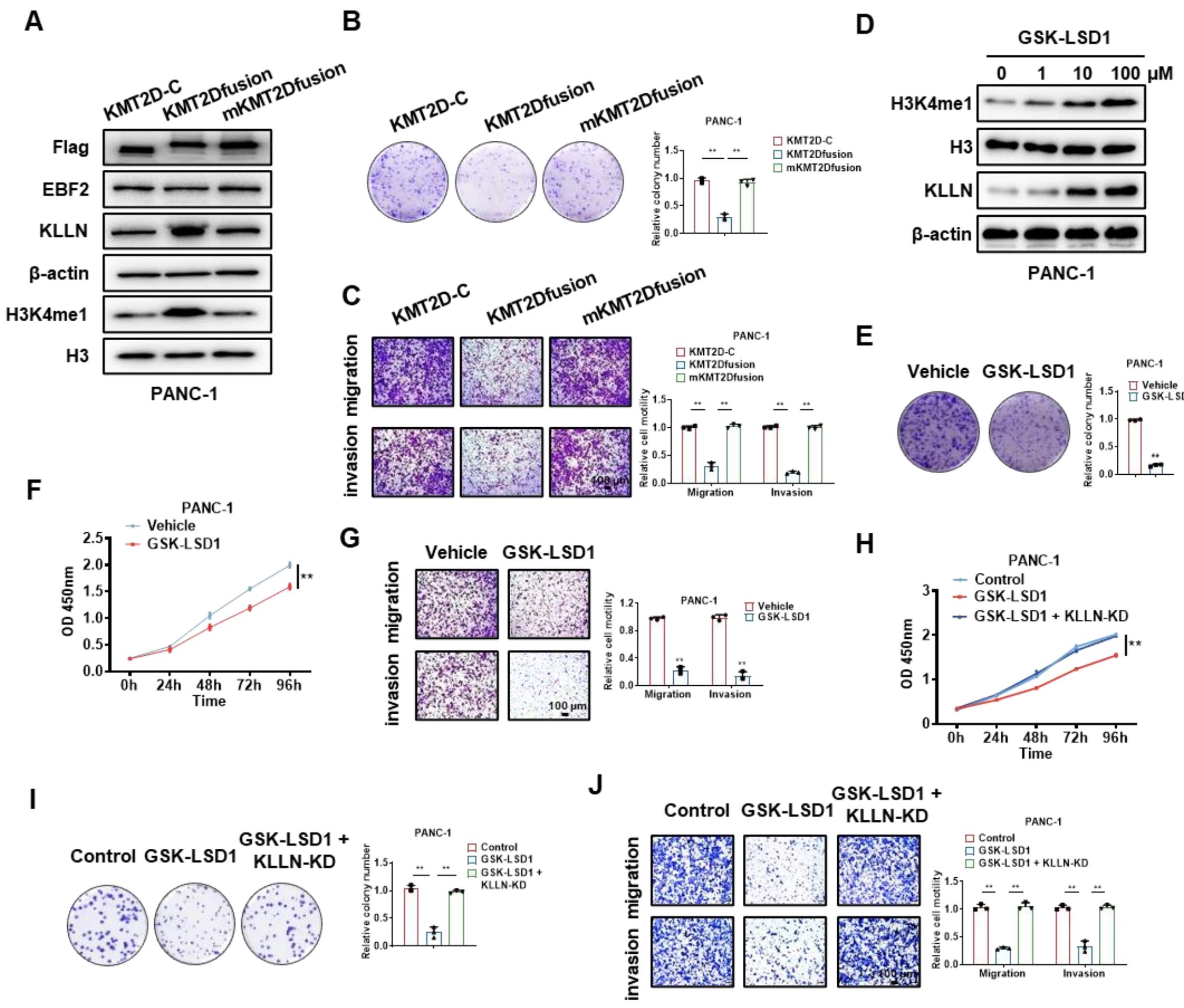

5. Figure S4. GSK-LSD1 inhibits the proliferation, migration and invasion of PDAC cells. A) Western blot analysis of KLLN and H3K4me1 levels in PANC-1 cells transfected with KMT2D-C, KMT2D$_{fusion}$ and mKMT2D$_{fusion}$. B-C) Colony-formation (B) and Transwell (C) assays of PANC-1 cells transfected with KMT2D-C, KMT2D$_{fusion}$ and mKMT2D$_{fusion}$. Data in B-C are presented as mean ± SEM, $^{**}P<0.01$ by one-way ANOVA test. D) Western blot analysis of H3K4me1 and KLLN levels in PANC-1 cells treated with 0, 1, 10 or 100μM GSK-LSD1. E-G) Colony-formation (E), CCK-8 (F) and Transwell (G) assays of PANC-1 cells treated with Vehicle and GSK-LSD1. Data in E-G are presented as mean ± SEM, $^{**}P<0.01$ by Student's *t*-test (E and G), two-way ANOVA test (F). H-J) CCK-8 (H), colony-formation (I) and Transwell (J) assays were used to detect the proliferation, migration and invasion of control, GSK-LSD1 and GSK-LSD1 + KLLN PANC-1 cells. Data in H-J are presented as mean ± SEM, $^{**}P<0.01$ by two-way ANOVA test (H), one-way ANOVA test (I-J).

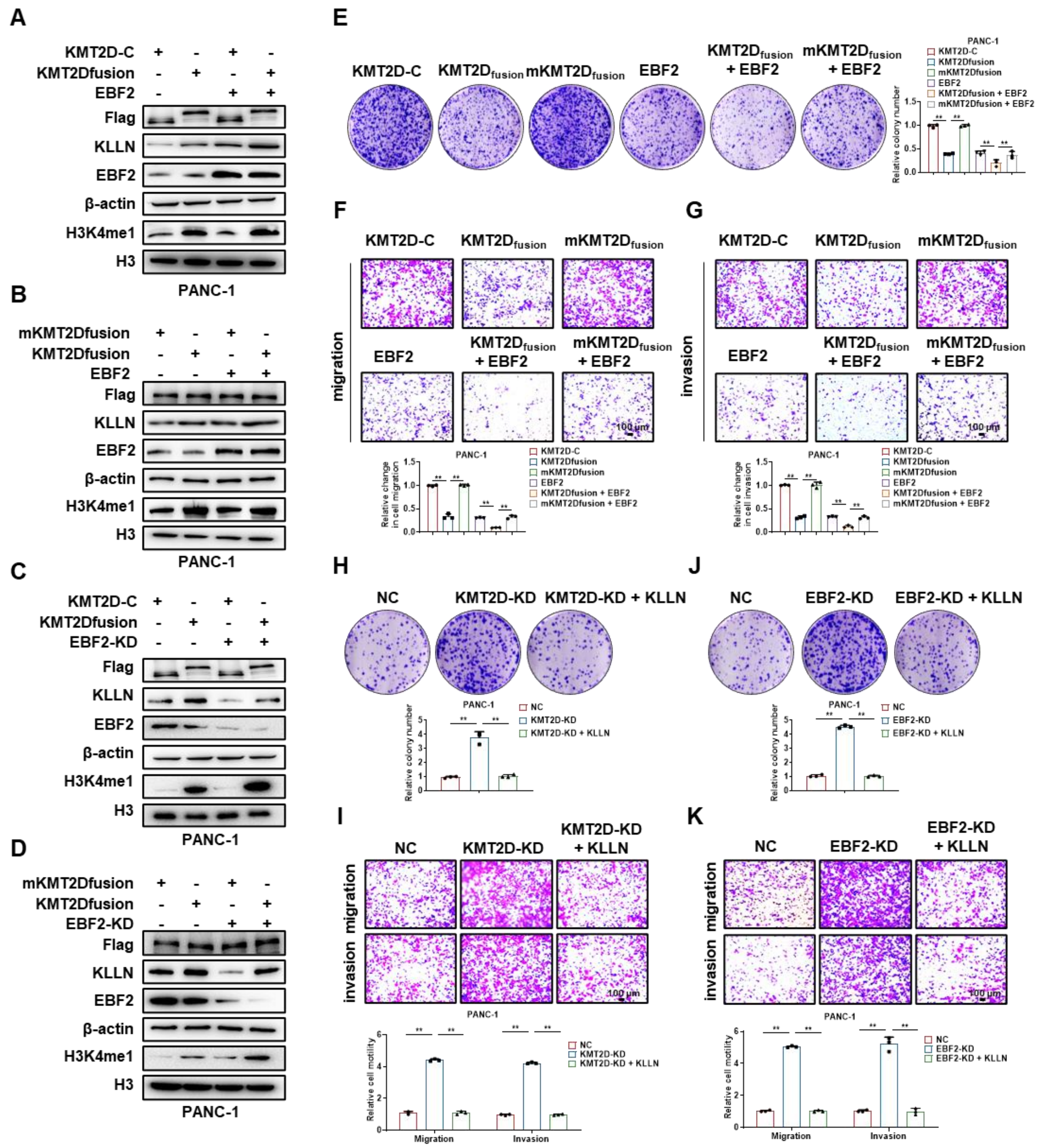

6. Figure S5. KMT2D and EBF2 cooperate to activate the expression of KLLN. A) Western blot analysis of KLLN and H3K4me1 levels in PANC-1 cells transfected with KMT2D-C, $KMT2D_{fusion}$ or EBF2. B) Western blot analysis of KLLN and H3K4me1 levels in PANC-1 cells transfected with $mKMT2D_{fusion}$, $KMT2D_{fusion}$ or EBF2. C) Western blot analysis of KLLN and H3K4me1 levels in PANC-1 cells transfected with KMT2D-C, $KMT2D_{fusion}$ or EBF2-KD. D) Western blot analysis of KLLN and H3K4me1 levels in PANC-1 cells transfected with $mKMT2D_{fusion}$, $KMT2D_{fusion}$ or EBF2-KD. β-actin and H3 were used as protein loading control. E-G) Colony-formation (E), migration (F) and invasion (G) assays of PANC-1 cells treated with KMT2D-C, $KMT2D_{fusion}$, $mKMT2D_{fusion}$, EBF2, $KMT2D_{fusion}$ + EBF2, $mKMT2D_{fusion}$ + EBF2. H-I) Colony-formation (H), Transwell (I) assays of PANC-1 cells transfected with NC, KMT2D-KD or KMT2D-KD +

KLLN. J-K) Colony-formation (J), Transwell (K) assays of PANC-1 cells transfected with NC, EBF2-KD or EBF2-KD + KLLN. Data in E-K are presented as mean ± SEM, $^{**}P<0.01$ by one-way ANOVA test.

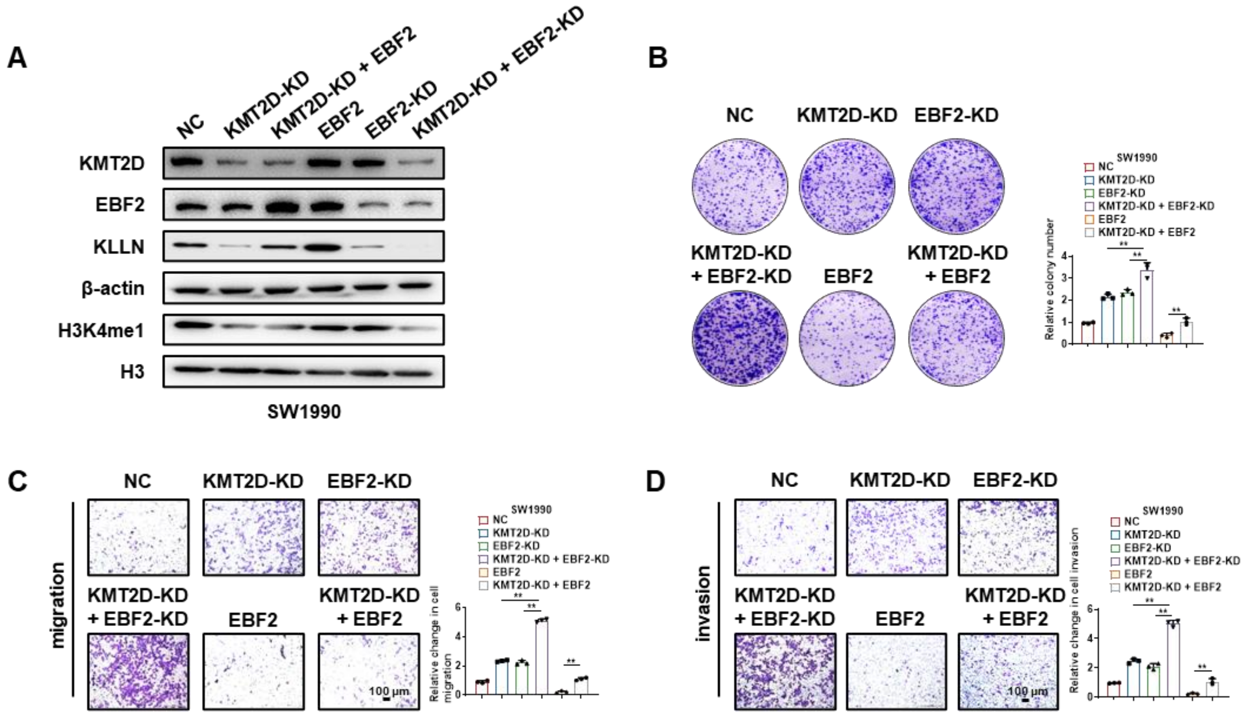

7. Figure S6. EBF2 and KMT2D cooperate to inhibit the proliferation, migration and invasion of PDAC cells *in vitro*. A) Western blot analysis of KLLN and H3K4me1 levels in NC, KMT2D-KD, KMT2D-KD+EBF2, EBF2, EBF2-KD or KMT2D-KD+EBF2-KD SW1990 cells. B-D) Colony-formation (B) and migration (C) and invasion (D) assays of PANC-1 cells with NC, KMT2D-KD, KMT2D-KD + EBF2, EBF2, EBF2-KD or KMT2D-KD+EBF2-KD. Data in B-D are presented as mean ± SEM, $^{**}P < 0.01$ by one-way ANOVA test.
